# Peripheral artery disease, antithrombotic treatment and outcomes in European and Asian patients with atrial fibrillation: analysis from two prospective observational registries

**DOI:** 10.1186/s12916-024-03792-3

**Published:** 2024-12-02

**Authors:** Davide Antonio Mei, Giulio Francesco Romiti, Tommaso Bucci, Bernadette Corica, Jacopo Francesco Imberti, Niccolò Bonini, Marco Vitolo, Alena Shantsila, Hung-Fat Tse, Tze-Fan Chao, Giuseppe Boriani, Marco Proietti, Gregory Y. H. Lip

**Affiliations:** 1 Liverpool Centre for Cardiovascular Science at University of Liverpool, Liverpool John Moores University and Liverpool Heart & Chest Hospital, Liverpool, UK; 2 Cardiology Division, Department of Biomedical, Metabolic and Neural Sciences, Italy University of Modena and Reggio Emilia, Policlinico Di Modena, Modena, Italy; 3 Clinical and Experimental Medicine PhD Program, University of Modena and Reggio Emilia, Modena, Italy; 4 Department of Translational and Precision Medicine, Sapienza, University of Rome, Rome, Italy; 5 Department of General and Specialized Surgery, Sapienza University of Rome, Rome, Italy; 6 Division of Cardiology, Department of Medicine, School of Clinical Medicine, Queen Mary Hospital, the University of Hong Kong, Hong Kong SAR, China; 7 Institute of Clinical Medicine, and Cardiovascular Research Center, National Yang Ming Chiao Tung University, Taipei, Taiwan; 8 Division of Cardiology, Department of Medicine, Taipei Veterans General Hospital, Taipei, Taiwan; 9 Department of Clinical Sciences and Community Health, University of Milan, Milan, Italy; 10 Division of Subacute Care, IRCCS Istituti Clinici Scientifici Maugeri, Milani, Italy; 11 Department of Clinical Medicine, Aalborg University, Aalborg, Denmark

**Keywords:** Atrial fibrillation, Peripheral artery disease, Oral anticoagulation, Europe, Asia

## Abstract

**Background:**

In patients with atrial fibrillation (AF), the impact of peripheral artery disease (PAD) on oral anticoagulant (OAC) therapy use and the risk of outcomes remains unclear.

**Objective:**

To analyse the epidemiology of PAD in a large cohort of European and Asian AF patients, and the impact on treatment patterns and risks of adverse outcomes.

**Methods:**

We analysed AF patients from two large prospective observational registries. OAC prescription and risk of outcomes were analysed according to the presence of PAD, using adjusted Logistic and Cox regression analyses. The primary outcome was the composite of all-cause death and major adverse cardiovascular events (MACE). Interaction analyses were also performed.

**Results:**

Fifteen-thousand-four-hundred-ninety-seven patients with AF (mean age 68.9, SD 11.6 years; 38.6% female, 30% from Asia) were included in the analysis. PAD was found in 941 patients (6.1%), with a higher prevalence among European individuals compared to Asian (8.1% vs 1.2%, *p* < 0.001). On logistic regression analysis, European patients had sixfold higher odds of presenting with PAD compared with Asians (OR 6.23, 95% CI 4.75–8.35).

After adjustments, PAD was associated with lower use of OAC (OR: 0.59, 95% CI: 0.50–0.69). On Cox regression analysis, PAD was associated with a higher risk of the primary composite outcome (HR 1.28, 95% CI: 1.08–1.52) and all-cause death (HR 1.40, 95% CI: 1.16–1.69). A significant interaction was observed between PAD and age, with higher effects of PAD found in younger patients (< 65 years) for the risk of the primary outcome (*p*_int_ = 0.014).

**Conclusions:**

In patients with AF, PAD is associated with lower use of OAC and a higher risk of adverse outcomes, with a greater risk seen in younger patients.

**Supplementary Information:**

The online version contains supplementary material available at 10.1186/s12916-024-03792-3.

## Background

Peripheral artery disease (PAD) and atrial fibrillation (AF) are both associated with a higher risk of cardiovascular and cerebrovascular events and mortality [1, 2]. In observational studies, the simultaneous presence of AF and PAD has been previously linked with an additive risk of adverse events [3]. Furthermore, the prevalence of PAD has increased globally among younger patients [4]. These individuals tend to have an aggressive disease course, accompanied by a high burden of other cardiovascular risk factors, thus increasing the probability of adverse events.

The detrimental effects of PAD in patients with AF have been previously recognized, being associated with higher thromboembolic risk and also included in stroke risk stratification scores [5]. While PAD usually requires antiplatelet therapy as thromboprophylaxis, patients with concomitant AF and PAD are recommended stroke prevention with oral anticoagulants (OAC) [6, 7]. Nonetheless, previous reports have shown significant rates of inappropriate OAC prescription [8], leading in turn to a worse prognosis. In the past, the co-existence of stable vascular disease and AF led to inappropriate co-prescription of antiplatelet (ATP) and OAC therapy, despite guidelines recommending the use of OAC treatment alone [6, 9]. Also, other pharmacological treatments may play a role in this complex interplay. A previous report from the EORP-AF Pilot registry showed that disease modifier treatment (statin, ACE-I and calcium channel blockers) may modulate the risk of adverse events among patients with AF [10]. However, there is still a lack of solid data regarding the management of patients with AF and PAD as well as on the complex relationship between PAD, AF and other cardiovascular risk factors. Similarly, data on potential geographical differences in the epidemiology of PAD in patients with AF are currently limited.

In this study, we aimed to investigate the epidemiology of PAD in patients with AF, and its relationship with antithrombotic management, as well as risk of adverse cardiovascular events, in two large cohorts of European and Asian patients.

## Methods

### Study design

We included patients with AF from two large, prospective observational registries from Europe and Asia. Details on the studies’ design, baseline characteristics and primary results have been previously published [11, 12]. Briefly, both registries enrolled consecutive adult patients (> 18 years old) with an ECG-documented episode of AF in the 12 months before inclusion. The type of AF was classified according to European Guidelines [6] (i.e. first-detected AF, paroxysmal AF, persistent AF, long-standing persistent AF, and permanent AF) and was defined by the investigator at baseline. All patients enrolled provided written informed consent.

In the European registry (EURObservational Research Programme in Atrial Fibrillation General Long-Term Registry), patients were enrolled in 250 participating centres across 27 countries between October 2013 and September 2016, with a pre-planned 2-year follow-up. The centres were from all over Europe, providing a comprehensive representation of the European AF population. The study protocol was approved for each country and for each enrolling site by the National Coordinators’ main institutions. The study was performed according to the European Union Note for Guidance on Good Clinical Practice CPMP/ECH/135/95 and the Declaration of Helsinki. The Asian registry (Asia–Pacific Heart Rhythm Society Atrial Fibrillation registry) enrolled patients in 52 centres across five countries, in particular from Southeast Asia. It was established in late 2015. Patients were enrolled until early 2017 and followed for 1 year; the study protocol was approved by the local ethics committees.

At baseline, investigators collected data regarding demographics, comorbidities, and pharmacological treatment. Data were collected using a standardized electronic case report form (eCRF). The same eCRF with the same variables was used to collect data for the European and Asian registries. To make it easier to follow the manuscript, we refer to patients from the European registry as “European patients” and those from the Asian registry as “Asian patients” throughout the text. This terminology reflects the registry from which the data were derived rather than the exact geographic origin of the patients. Data regarding race/ethnicity distribution in the two registries are reported in the Additional file 1: Table S1.

PAD diagnosis was established by investigators at site level. The presence of PAD was defined by a positive history of any of the following: intermittent claudication, previous arterial surgery, percutaneous intervention or thrombosis of the abdominal or thoracic aorta and lower extremity arteries. This assessment was performed by any physician during the clinical assessment and/or via medical records, if available. No information regarding the severity of PAD was routinely collected.

For this analysis, we included AF patients with available data regarding the presence of PAD reported at baseline. We then considered two groups: (i) patients with a no recorded history of PAD (“No PAD” group), and (ii) patients with a recorded history of PAD (“PAD” group).

### Pharmacological treatment

Data regarding pharmacological treatment at discharge were collected by the investigators for each patient enrolled. To assess the relationships between PAD and antithrombotic therapy, we considered: (i) OAC therapy (either vitamin K antagonist [VKA] or Non-vitamin K oral anticoagulant [NOAC] [13]); (ii) NOAC vs VKA; (iii) any antiplatelet therapy (APT, defined as treatment with aspirin, clopidogrel, ticagrelor, prasugrel or ticlopidine]); (iv) combination therapy of OAC + APT.

We additionally evaluated the relationship between PAD and therapies used to treat other cardiovascular disorders: (i) ACE inhibitors (ACE-i) or angiotensin receptor blockers (ARBs); (ii) mineralocorticoid receptor antagonists (MRAs); (iii) calcium channel blockers (CCBs); and (iv) statins. Lastly, we evaluated the association between PAD and the prescription of different therapies commonly prescribed in AF patients: (i) beta-blockers; (ii) non-dihydropyridine calcium channel blockers (non-DHP CCBs); (iii) digoxin; (iv) class IC antiarrhythmic drugs (IC AADs) defined as therapy with flecainide or propafenone; (v) class III antiarrhythmic drugs (III AADs) defined as therapy with amiodarone or dronedarone or sotalol. We also evaluated aggregated pharmacological treatments subdivided as follows: (i) any rate control therapy (defined as therapy with beta-blockers or non-DHP CCBs or digoxin) and (ii) any AADs (defined as therapy with IC AADs or III AADs).

### Follow-up and adverse outcomes

As per the original design of the two studies, patients enrolled in the European registry were followed for 2 years, while those recruited in the Asian registry were followed for 1 year. Incidence of major adverse events was collected by the investigators during follow-up. Major adverse events were as follows: (i) all-cause death; (ii) cardiovascular (CV) death; (iii) any acute coronary syndrome (ACS); (iv) any thromboembolic (TE) events (defined as a composite of stroke, transient ischemic attack [TIA], and peripheral embolism); and (v) major bleeding (which included intracranial and/or extracranial major bleeding).

For this analysis, we defined our primary outcome as a composite of all-cause death, any ACS, and any TE. As exploratory secondary outcomes, we evaluated: (i) all-cause death; (ii) a composite endpoint of major adverse cardiovascular events (MACE) (defined as CV-death, any ACS, or any TE); and (iii) major bleeding.

### Statistical analysis

Continuous variables were reported as median and interquartile range (IQR), and comparison between groups was performed using the Kruskal–Wallis test. Categorical variables were reported as counts and percentages and compared using the chi-square test.

We used multivariable logistic regression to assess the variables associated with the presence of PAD. Covariates included in the model were the components of the CHA_2_DS_2_-VASc score (age used as a categorical variable and then modelled as a continuous variable using a restricted cubic spline with 3 knots, sex, hypertension, diabetes mellitus, coronary artery disease, heart failure, and previous TE), and the registry of enrolment (Europe or Asia). A second model, which additionally included the type of AF and European Heart Rhythm Association (EHRA) score, was used to evaluate the association between PAD and antithrombotic treatments. Results were reported as odds ratio (OR), with 95% confidence interval (CI).

Kaplan–Meier curves were drafted to study the cumulative survival curves for the outcomes of the study, and survival distribution was tested for differences across the groups with the log-rank test. Patients included in the survival analysis were those with available data regarding the occurrence of the primary endpoint. To assess the potential influence of missing data, we compared the populations included and excluded from the survival analysis.

Multivariable Cox regressions were used to investigate the relationship between the presence of PAD and the outcomes of the study. We evaluated different multivariable Cox models:

- Model 1: adjusted for age, sex, registry, type of AF and EHRA score;
- Model 2: same variables included in model 1 plus the remaining components of the CHA_2_DS_2_-VASC score (hypertension, diabetes mellitus, CAD, previous TE, HF);
- Model 3: same variables included in model 2, plus treatment with OAC and/or statin.

We also performed several subgroup analyses, according to different age groups (< 65 years; 65–80 years; > 80 years), sex, type of registry, low vs high thromboembolic risk (defined as CHA_2_DS_2_-VASc ≥ 3 for male and ≥ 4 for female) and bleeding risk (defined as HAS-BLED ≥ 3), for both the probability of being prescribed different antithrombotic treatments and the risk of the primary composite outcome of the study. We also assessed the interplay between PAD and OAC and statin treatment by performing an interaction analysis for the risk of outcome. Finally, we also evaluated the interaction between age, modelled as a continuous, non-linear variable, and PAD on the risk of outcomes of the study, using a restricted cubic spline with 4 knots. Restricted cubic splines allow us to test the hypothesis that the relationship between age and outcomes is not linear. In restricted cubic splines, the range of values for the independent variable (age) is split into segments, with knots defining the end of one segment and the start of the next. A reference value of 65 years was used to compare the association of age with the risk of outcomes.

Results were reported as hazard ratio (HR) with 95% confidence interval (CI). A two-sided *p*-value < 0.05 was considered statistically significant. All analyses were performed using R 4.0.3 (R Core Team 2020, Vienna, Austria).

## Results

### Baseline characteristics

Among the 15,762 AF patients enrolled in the two registries, 15,497 (mean age 68.9, SD 11.6 years; 38.6% female, 30.0% Asian) with complete data regarding the presence of PAD were included in the analysis. Overall, PAD was found in 941 patients, yielding a total prevalence of 6.1%. PAD was significantly more prevalent among European compared to Asian individuals (8.1% vs 1.2%, *p* < 0.001).

Table 1 reports the baseline characteristics of the two cohorts. Overall, patients with AF and PAD were more likely to have permanent AF (43.5% vs 28.1%, *p* < 0.001), symptomatic AF (EHRA score III–IV, 18.9% vs 15.2%, *p* = 0.002), cardiovascular/non-cardiovascular comorbidities, as well as higher overall thromboembolic and bleeding risks.

**Table 1:** Baseline characteristics in atrial fibrillation patients according to the presence of peripheral artery disease

	No PAD *N* = 14,556 (93.9%)	PAD *N* = 941 (6.1%)	*p*
**Age (years) (median (IQR])**	70.00 (62.00, 77.00)	74.00 (67.00, 79.00)	** < 0.001**
**Female, *N* (%)**	5638/14556 (38.7)	348/941 (37.0)	0.301
**BMI (median (IQR))**	26.60 (23.90, 30.00)	27.30 (24.60, 30.90)	** < 0.001**
**Type of atrial fibrillation, *N* (%)**			** < 0.001**
First diagnosed	1924/14,366 (13.4)	114/930 (12.3)	
Paroxysmal	4518/14,366 (31.4)	218/930 (23.4)	
Persistent	3012/14,366 (21.0)	146/930 (15.7)	
Long-standing persistent	877/14,366 (6.1)	47/930 (5.1)	
Permanent	4035/14,366 (28.1)	405/930 (43.5)	
**Cardiovascular comorbidities**			
Hypertension, *N* (%)	8796/14,441 (60.9)	679/937 (72.5)	** < 0.001**
Diabetes, *N* (%)	3242/14,430 (22.5)	343/936 (36.6)	** < 0.001**
Dyslipidemia, *N* (%)	5459/14,076 (38.8)	545/923 (59.0)	** < 0.001**
Coronary artery disease, *N* (%)	3412/13,937 (24.5)	431/885 (48.7)	** < 0.001**
Heart failure, *N* (%)	4630/14,426 (32.1)	535/931 (57.5)	** < 0.001**
LVEF (%) (median (IQR))	58.00 (50.00, 63.00)	55.00 (44.00, 61.00)	** < 0.001**
Previous thromboembolic events, *N* (%)	1526/14,435 (10.6)	217/932 (23.3)	** < 0.001**
**Other comorbidities**			
Chronic kidney disease, *N* (%)	1436/14,496 (9.9)	254/929 (27.3)	** < 0.001**
Malignancy (current or prior), *N* (%)	1046/14,500 (7.2)	126/934 (13.5)	** < 0.001**
Previous hemorrhagic events, *N* (%)	854/14,443 (5.9)	64/929 (6.9)	0.252
Anaemia, *N* (%)	789/14,531 (5.4)	122/937 (13.0)	** < 0.001**
**CHA2DS2-VASc (median (IQR))**	3.00 (2.00, 4.00)	5.00 (4.00, 6.00)	** < 0.001**
**HAS-BLED (median (IQR))**	1.00 (1.00, 2.00)	2.00 (1.00, 3.00)	** < 0.001**
**EHRA score III–IV, *N* (%)**	2207/14,556 (15.2)	178/940 (18.9)	**0.002**
**EHRA score, *N* (%)**			** < 0.001**
I	7408/14,556 (50.9)	501/940 (53.3)	
II	4941/14,556 (33.9)	261/940 (27.8)	
III	1965/14,556 (13.5)	149/940 (15.9)	
IV	242/14,556 (1.7)	29/940 (3.1)	

*BMI* body mass index, *CKD* chronic kidney disease, *EHRA* European Heart Rhythm Association, *IQR* interquartile range, *LVEF* left ventricular ejection fraction, *N* number, *TE* thromboembolic events

We also report the distribution of baseline characteristics categorized for the registry of enrollment and the presence of PAD in Additional File 1: Table S2. European patients with PAD were more likely to have permanent AF compared to Asian as well as a higher prevalence of CAD and HF. Conversely, Asian patients with PAD were less symptomatic and more often diagnosed with chronic kidney disease and previous TE.

### Factors associated with presence of PAD

**Fig. 1:**
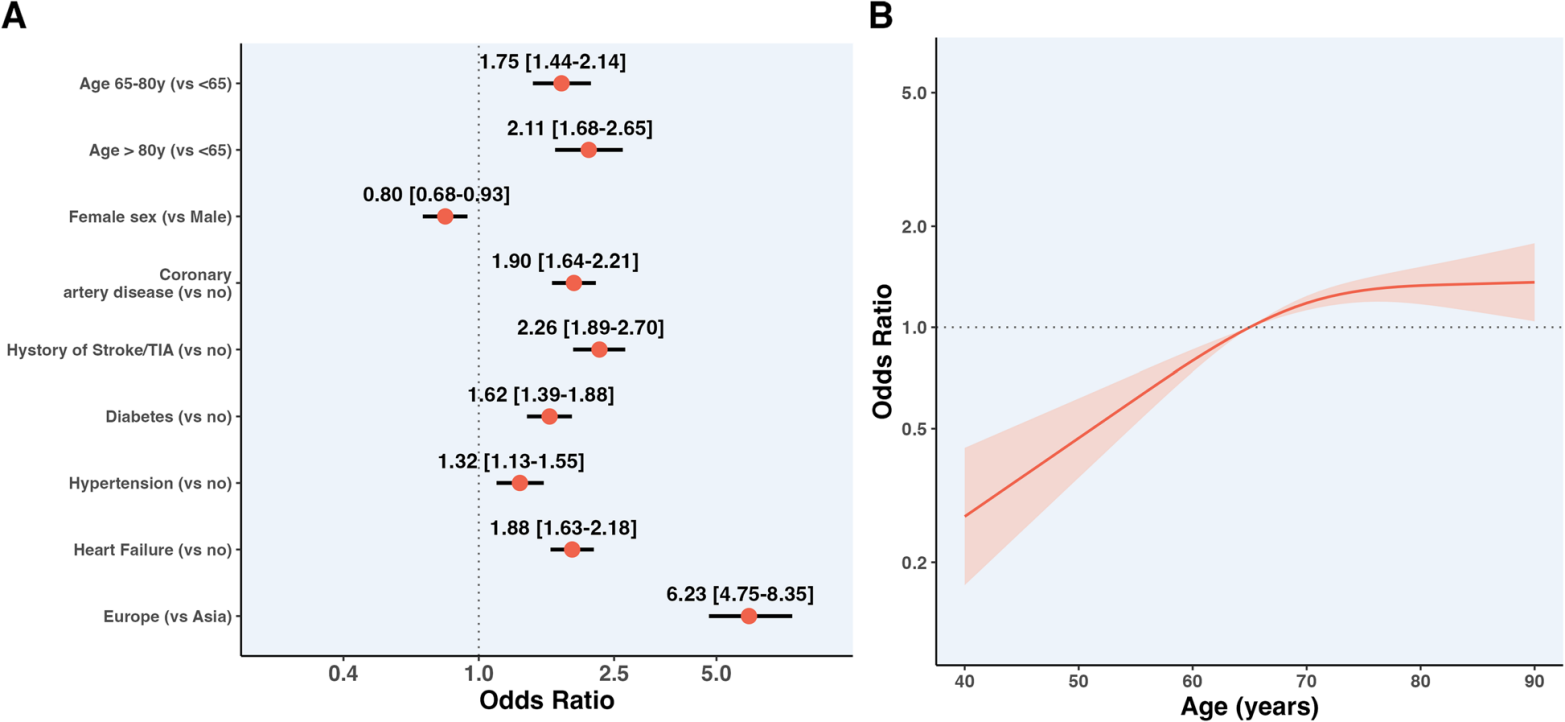
Clinical factors associated with the presence of PAD. **A** Logistic regression analysis displaying the different odds for the presence of PAD for the categorical variables included in the model. **B** Logistic regression modelled as a spline curve for age used as a continuous variable. Legend. Models were adjusted for the components of the CHA_2_DS_2_-VASc score and the registry of enrolment. Where not specified. In panel **A**, values at the right of the dotted line indicate increased odds of PAD, while at the left reduced odds

Results of logistic regression analyses on the association between clinical factors and odds of PAD at baseline are reported in Fig. 1, panel A. Compared to patients enrolled in Asia, those enrolled in Europe had sixfold higher odds of presenting with PAD (OR 6.23, 95% CI 4.75–8.35).

Similarly, other comorbidities (CAD, history of Stroke/TIA, diabetes, hypertension, and HF) and increasing age were associated with higher odds of PAD. Conversely, female sex was associated with lower odds of PAD at baseline. When we analysed the associations between age modelled as a continuous variable, and odds of PAD at baseline, there was a non-linear relationship, with odds of PAD increasing almost linearly until 70 years, plateauing afterwards. (Fig. 1, panel B).

### Associations between PAD and pharmacological treatments

**Fig. 2:**
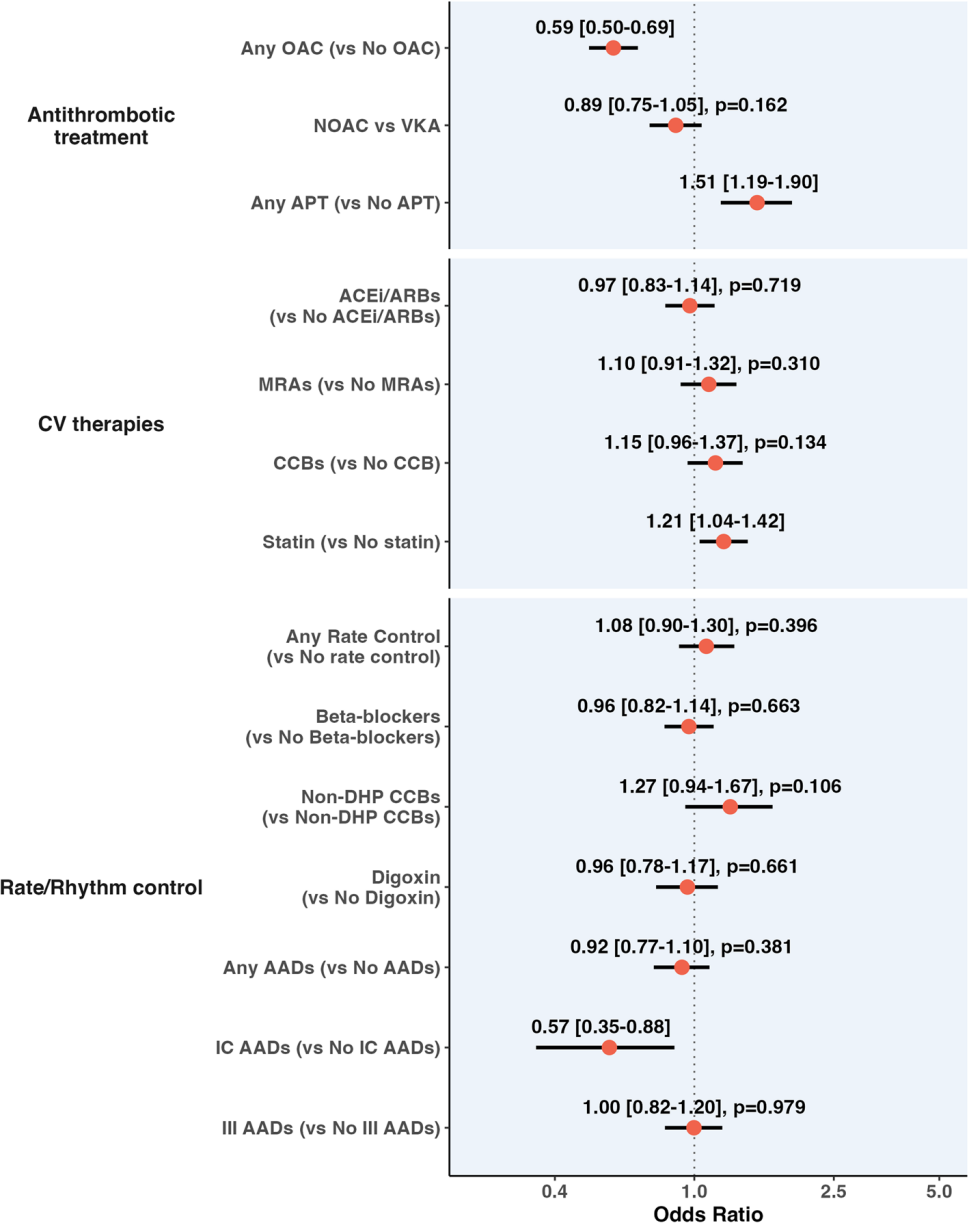
Logistic regression analysis investigating the association between PAD and different pharmacological therapies. Legend. AAD, antiarrhythmic drug; ARB, angiotensin receptor blockers; APT, antiplatelet; CCB, calcium channel blocker; MRA, mineral receptor antagonist; NOAC, non-vitamin K; oral anticoagulant; non-DHP, non-dihydropyridine; PAD, peripheral artery disease; VKA, vitamin K antagonist. Adjusted for the components of the CHA_2_DS_2_-VASc score, registry of enrolment type of AF and EHRA score. Values at the right of the dotted line indicate increased odds of being prescribed with the treatment, while at the left reduced odds

Pharmacological treatments according to PAD are reported in Additional File 1: Table S3, while the results of the logistic regression analysis for the association between PAD and the use of pharmacological treatments are reported in Fig. 2.

Patients with PAD had lower odds of receiving OACs (OR: 0.59, 95% CI: 0.50–0.69), while no statistically significant differences were observed for OAC type. Conversely, patients with PAD had a higher probability of being prescribed any APT therapy (OR: 1.51, 95% CI: 1.19–1.90) and OAC + APT (OR: 1.47, 95% CI 1.21–1.78). Patients with PAD were associated with a higher likelihood of receiving treatment with statins and lower odds of treatment with class IC AADs. No significant differences were observed for the other treatments analysed (Fig. 2).

We also analysed the association between PAD and the use of antithrombotic drugs according to different subgroups. There was a significant interaction effect for sex, with female patients showing lower odds of receiving OAC than males (OR 0.43, 95% CI 0.33–0.56 vs OR: 0.70, 95% CI 0.57–0.85, respectively; *p*_int_ = 0.004, Additional File 1: Fig. S1). Asian patients with PAD tended to be prescribed OAC + APT more often compared to Europeans (OR 3.20, 95% CI 1.55–6.61 vs OR 1.40, 95% CI 1.14–1.71, respectively; *p*_int_ = 0.031). The high bleeding risk subgroup showed lower odds of being prescribed with OAC + APT compared to those with low risk (OR 1.09, 95% CI 0.81–1.48 vs OR 1.63, 95% CI 1.27–2.10, respectively; *p*_int_ = 0.045). No significant interaction effects were observed according to thromboembolic risk, or between European and Asian cohorts; similarly, there were no significant subgroup differences for NOAC vs. VKA and APT use (Additional File 1: Fig. S1).

### Follow-up and risk of the adverse outcomes according to PAD

The survival analysis included 13,606 (88%) patients with available data regarding the occurrence of the primary composite outcome. Additional File 1: Table S4 shows the differences in baseline characteristics between the groups included and not included in the survival analysis. Patients excluded were younger, had lower BMI values, a higher proportion of dyslipidaemia, and a lower incidence of malignancy. No other significant differences were found.

After a median follow-up of 690 days (IQR 365–735), a total of 1664 (12.2%) primary composite outcome events occurred. Crude outcome rates and cumulative incidence were higher in patients with PAD (Additional File 1: Table S5 and Additional File 1: Fig. S2; log-rank *p* < 0.001).

**Fig. 3:**
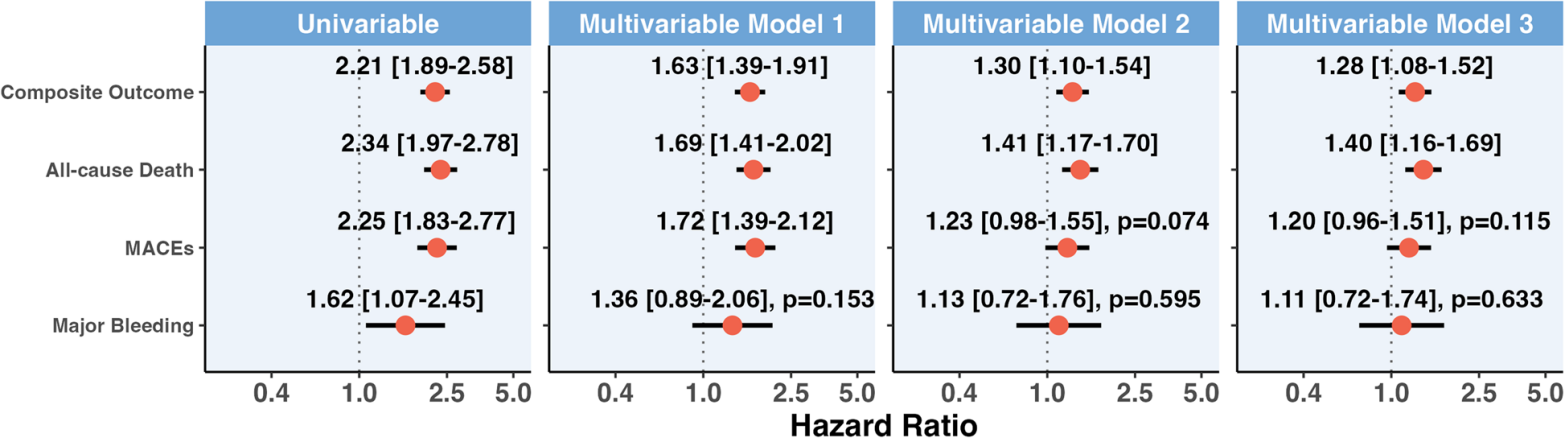
Results of univariable and different multivariable Cox regression analysis for the relationship between PAD and outcomes of the study. Legend. The composite outcome included all-cause death and MACE; MACE, major adverse cardiovascular events. Model 1: adjusted for Age, sex, type of atrial fibrillation, EHRA score, type of registry. Model 2: as per Model 1 + hypertension, diabetes, heart failure, previous thromboembolism, coronary artery disease. Model 3: as per Model 2 + statin and OAC therapy. Values at the right of the dotted line indicate an increased hazard of adverse events, while at the left reduced hazard

Univariable and multivariable Cox regression models are reported in Fig. 3. In all models, PAD was associated with increased risk of the primary composite outcome, which was consistent across the 3 multivariable models performed (model 3 adjusted hazard ratio (aHR) 1.28, 95% CI: 1.08–1.52); similar results were observed for all-cause death (model 3 aHR 1.40, 95% CI: 1.16–1.69). We also observed a non-significant trend for an increased risk of MACE in patients with PAD (model 3 aHR 1.20, 95% CI: 0.96–1.51, *p* = 0.115), while no statistically significant differences were observed for the risk of major bleeding. Distribution of missing data for variables included in the models is reported in Additional File 1: Table S6.

### Subgroup analyses

**Fig. 4:**
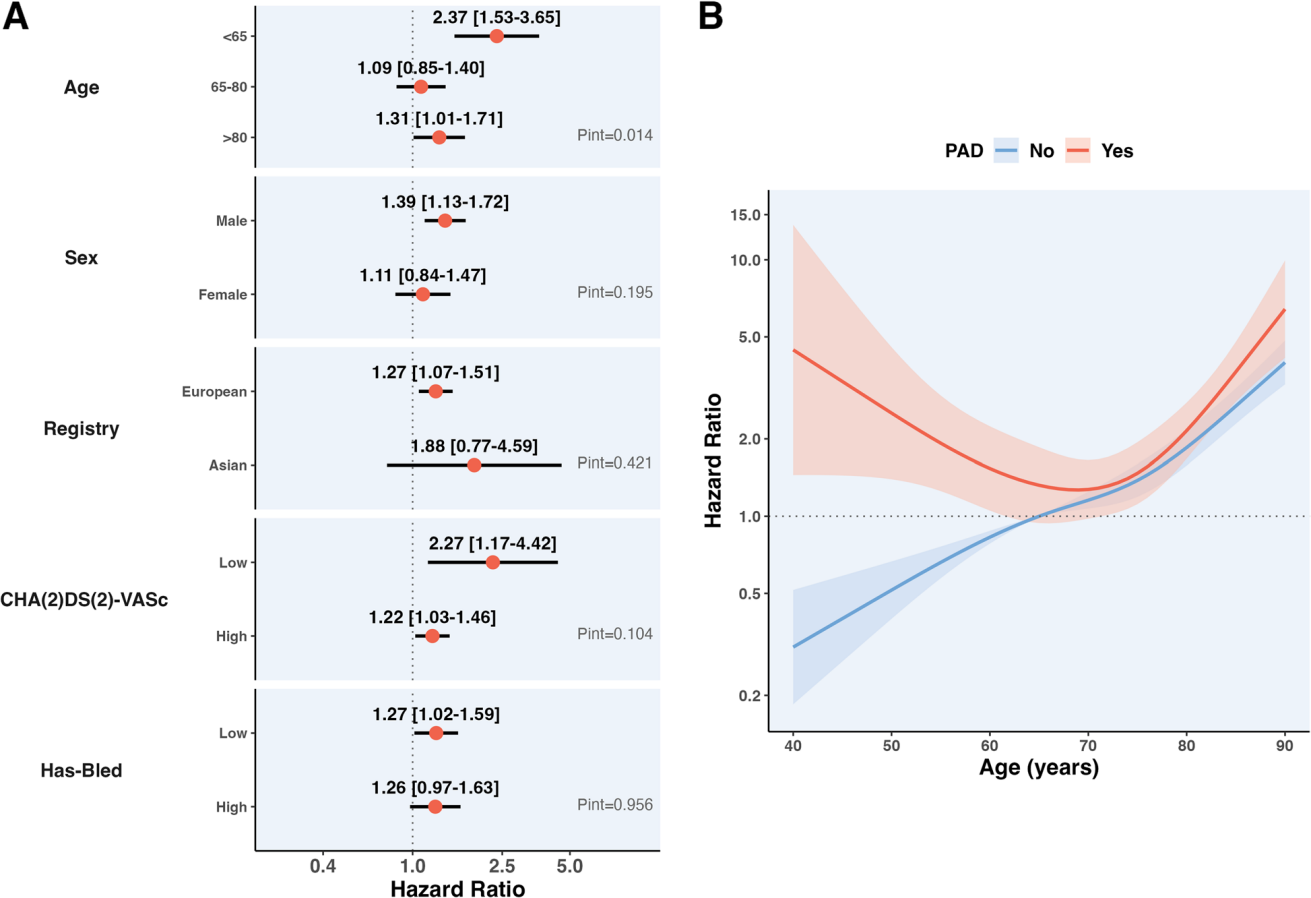
Subgroup analysis for the primary endpoint of the study. **A** Interaction analysis for the risk of the primary composite outcome among different subgroups considered. **B** Interaction analysis plotted as a restricted cubic spline using age as a continuous variable. Legend. Adjustments are as per Model 3. Values at the right of the dotted line indicate an increased hazard of adverse events, while at the left reduced hazard

Subgroup analyses for the primary composite outcome are shown in Fig. 4, panel A. No significant interaction was found for the risk of the primary outcome in patients with PAD according to sex, registry of enrolment, high vs low CHA_2_DS_2_-VASc and HAS-BLED scores.

In younger patients (< 65 years), PAD was associated with a higher magnitude of risk increase for the primary composite outcome (aHR: 2.37, 95% CI: 1.53–3.65), compared to that observed in patients 65–80 years old and ≥ 80 years (*p*_int_ = 0.014).

Consistent results were observed when we analysed the interaction between PAD and age, modelled as a restricted cubic spline. In patients with PAD, the risk of the primary outcome followed a J-curve across age, while patients without PAD showed an approximately linear relationship (Fig. 4, panel B; *p*_int_ < 0.001).

In relation to the two registries, Asian patients with PAD had lower incidence rates (IR) of composite outcome events compared to Europeans (IR 11.02/100 person-years, 95% CI 3.58–25.71 vs IR 16.27/100 person-years, 95% CI 14.14–18.62; Additional File 1: Table S7).

Regarding the pharmacological treatment (Additional File 1: Table S8), we found a non-significant interaction effect considering PAD and OAC therapy (aHR for no OAC 1.51 95% CI 1.08–2.12 vs aHR for OAC 1.22 95% CI 1.00–1.48, *p*_int_ = 0.272). Conversely, we observed an interaction effect considering statin therapy (aHR for no statin 1.84, 95% CI 1.47–2.32, aHR for statin 0.92, 95% CI 0.72–1.18, *p*_int_ < 0.001).

## Discussion

In this analysis of a contemporary cohort of European and Asian AF patients, our principal results are as follows: (i) prevalence of PAD is considerable in patients with AF, particularly among Europeans, compared to Asians; (ii) cardiovascular comorbidities and male sex were associated with PAD, while age showed a non-linear relationship with PAD; (iii) the presence of PAD influenced pharmacological treatment, being associated with lower probability of being prescribed with OAC, especially among female patients; and (iv) PAD was associated with worse prognosis in patients with AF, with strength of association influenced by age.

The prevalence of PAD that we observed in our European and Asian AF patients is consistent with previous studies conducted both in the US and in Europe [14]. We also reported a higher prevalence in European patients than in Asian ones. These results are consistent with a previous meta-analysis, which reported that South Asian patients had a lower probability of being diagnosed with PAD (OR = 0.26, 95% CI 0.17 to 0.38, *p* < 0.001) [15]. While the precise reasons for these findings are unclear, underdiagnosis may explain these geographical differences [16], as well as the differences that we observed.

Diet may also play a role in these differences, as populations with diets rich in fibre and healthy fats, like traditional Asian diets, might have a lower risk of PAD, while Western diets high in processed foods and saturated fats could increase PAD prevalence [17].

Indeed, several cardiovascular comorbidities are significantly associated with the presence of PAD: hypertension, diabetes mellitus, HF, CAD and history of stroke/TIA are factors that have been already found to be associated with PAD both in the general population and in AF patients [18]. This association underlines the close relationship that connects risk factors with atherothrombotic burden and AF, resulting in a higher probability of being diagnosed with PAD [19]. In this scenario, ageing can bolster this detrimental interplay, being a well-recognized risk factor for PAD [19]. We further expanded these findings, showing a non-linear association between age and likelihood of reporting PAD, suggesting that age may play a role in the epidemiology of PAD, which can be influenced and modulated by the presence of other risk factors. Indeed, we show an inverse relationship between female sex and PAD, consistent with a lower burden of atherothrombotic disease observed in women seen in the general population [20].

We found that PAD is independently associated with different treatment patterns in patients with AF. Since our data are from real world experience, they reflect the common clinical practice in Europe and in Asia in the past decade. Indeed, for patients with AF and stable vascular disease, the co-prescription with an APT (even if the patient is already taking OAC), or the under-prescription of an OAC in favour of APT, are commonly found in real-world setting [21, 22]. These approaches have been associated with higher bleeding risk [23, 24], and with an inadequate stroke prevention [25], respectively. Indeed, in patients with AF and stable vascular disease (such as PAD), OAC monotherapy is the treatment of choice as suggested by guidelines on AF [6, 26], while single therapy with APT is not recommended by many guidelines worldwide [27]. The prediction of major bleeding events remains challenging in the real-world management of AF patients [28], thus strategies that minimize this risk seem preferable in clinical practice.

In our cohort of patients, PAD was associated with a higher risk of the primary composite outcome and of all-cause death. Previous results on the relationship between PAD and adverse cardiac events were controversial. In an ancillary analysis of the Atrial Fibrillation Follow-up Investigation of Rhythm Management (AFFIRM) trial on 4060 patients, PAD was significantly associated with higher mortality (HR 1.34, 95% CI 1.06–1.70, *p* = 0.016) [29], while in the EORP-AF Pilot registry, individuals with PAD had higher absolute rates of both cardiovascular and all-cause death, but PAD did not show an independent association with mortality in Cox regression analyses (HR 1.37, 95% CI 0.93–2.03, *p* = 0.110) [19]. In this context, our findings delve further into the role of PAD in influencing adverse cardiovascular events. The interaction analysis showed a non-significant interaction effect of OAC treatment according to PAD. This finding suggests that while OAC therapy is crucial for patients with AF, its impact on mitigating PAD-related adverse outcomes might not be as pronounced or direct as hypothesized [30]. Given that OAC therapy primarily aims to reduce the risk of stroke in AF, our results highlight that the relationship between PAD and adverse outcomes may be more complex and influenced by other factors not captured in our study.

Conversely, we observed a significant interaction effect between statin and PAD. This supports the role of statin in reducing CV events and mortality among individuals with PAD [7]. Taken together, these results suggest that the impact of PAD on the risk of adverse events may be attenuated by comprehensive pharmacological management.

We also found that PAD may exert a differential impact on prognosis in different subgroups of patients with AF: for example, we showed that PAD significantly influences the risk of adverse outcome, particularly in younger patients, while we did not observe any significant interaction for other subgroups.

Several hypotheses can explain these findings. First, PAD in younger individuals has already been associated with a higher risk of adverse events in the general population [4, 31]. Indeed, an early onset of the disease may reflect a higher thrombotic burden and a more aggressive disease, with a significant influence on cardiovascular outcomes. Second, as previously mentioned, patients with PAD were also significantly under-prescribed with OAC therapy. Even though we cannot assess a causal association with adverse events, it may be speculated that this trend may have negatively influenced the prognosis of younger individuals with PAD. Finally, the effect of PAD may appear magnified in younger patients with AF, considering their relatively lower baseline risk of adverse outcomes. In this regard, the contribution of PAD may appear diluted in older subjects, who present an intrinsic higher risk of adverse events due to the burden of other cardiovascular conditions as well as ageing.

Overall, our results have important clinical implications. We showed that patients with AF and PAD have a complex clinical profile, characterized by a higher atherothrombotic burden and a high risk of adverse outcomes, which negatively influences prognosis. Indeed, patients with AF and clinically complex phenotypes show worse outcomes and require further effort to improve prognosis [32, 33]. European and Asian guidelines on the management strategies of AF proposed the use of the “Atrial fibrillation Better Care” (ABC) pathway [21, 26], which has already been proven to reduce the risk of adverse events among clinically complex patients [34–37]. Indeed, patients with AF and PAD show a higher risk of thromboembolic events and therefore require lifelong OAC therapy for optimal stroke prevention. Our results highlight the fact that European and Asian AF patients with PAD may not have been properly managed with OAC in the last decade, thus requiring more efforts from physicians in order to adhere to a holistic and comprehensive management as encompassed by the ABC pathway. This approach may also potentially improve healthcare resource use and prognosis [36, 38].

## Study limitations

Our study has some limitations that should be acknowledged. This is a retrospective analysis of observational data, and possible bias may be present in the interpretation of our findings. We had no information on the severity or stage of PAD, and we cannot exclude a certain degree of underdiagnosis of PAD in our study, given that there was no mandatory routine assessment of its presence at enrolment. Second, even though we used multivariable analysis to adjust for possible confounders, we cannot exclude that other unaccounted factors may have influenced the findings observed. Given our sample size, we may have reduced power to evaluate differences, especially between subgroups. Moreover, our results were not adjusted for multiple comparisons, and therefore the results on the secondary outcomes should be interpreted with caution and regarded as exploratory and hypothesis-generating. Our cohorts of patients are representative of patients recruited among European and Asian countries, hence the results observed may not be representative of other geographical settings; moreover, we acknowledge that the differences found between the two registries may be related to the registries themselves and not driven by specific ethnic differences. The differences in follow-up lengths across the two registries, as per their original protocols, represent a limitation and warrant further caution in the interpretation of our results.

## Conclusions

In this large contemporary cohort of European and Asian AF patients, PAD was associated with a higher risk of the primary composite outcome of all-cause death and MACE. PAD was found to be associated with different antithrombotic treatment choices and under-prescription of OAC. The magnitude of the effect of PAD on major outcomes was significantly influenced by age, with higher effects exerted on younger patients.

## Supplementary Information

Additional file 1: Table S1. Ethnic distribution in the two registries. Table S2. Baseline characteristics according to the presence of PAD and Registry of enrolment. Table S3. Pharmacological treatment in the overall population accordingly to PAD. Table S4. Difference between patient included and not in survival analysis. Table S5. Outcome of the study according to PAD. Table S6. Missing data in variables included in the model, stratified by the presence of PAD. Table S7. Incidence rates for the outcomes of the study in patients with PAD, according to Registry of enrolment. Table S8. Subgroup analysis for the risk of primary outcome among subgroups of pharmacological treatment. Figure S1. Subgroup analysis for antithrombotic treatment. Figure S2. Kaplan–Meier curves for the endpoints of the study. Panel A: Composite outcome, Panel B: All-cause Death; Panel C: MACEs; Panel D: Any major bleeding. List of investigators.

## Data Availability

The data that support the findings of this study are available from the corresponding author, upon reasonable request, and after approval of all other co-investigators.

## Abbreviations

AADs: Antiarrhythmic drugs
ACS: Acute coronary syndrome
ACE-I: Angiotensin-converting enzyme inhibitor
APT: Antiplatelet therapy
ARB: Angiotensin receptor blocker
aHR: Adjusted hazard ratio
ATP: Antithrombotic therapy
CCBs: Calcium channel blockers
CI: Confidence interval
CV: Cardiovascular
DHP CCBs: Dihydropyridine calcium channel blockers
eCRF: Electronic case report form
EHRA: European Heart Rhythm Association
EURObservational: European Observational Research Programme
HF: Heart failure
IQR: Interquartile range
IR: Incidence rate
MACE: Major adverse cardiovascular events
MRAs: Mineralocorticoid receptor antagonists
NOAC: Non-vitamin K oral anticoagulant
OAC: Oral anticoagulant
OR: Odds ratio
PAD: Peripheral artery disease
SD: Standard deviation
TE: Thromboembolic
TIA: Transient ischemic attack
VKA: Vitamin K antagonist

## References

[1] Wolf PA, Abbott RD, Kannel WB. Atrial fibrillation as an independent risk factor for stroke: the Framingham Study. Stroke. 1991;22:983–8. 1866765 10.1161/01.str.22.8.983

[2] Grenon SM, Vittinghoff E, Owens CD, Conte MS, Whooley M, Cohen BE. Peripheral artery disease and risk of cardiovascular events in patients with coronary artery disease: insights from the Heart and Soul Study. Vasc Med. 2013;18:176–84. 23835937 10.1177/1358863X13493825 PMC4207208

[3] Anandasundaram B, Lane DA, Apostolakis S, Lip GY. The impact of atherosclerotic vascular disease in predicting a stroke, thromboembolism and mortality in atrial fibrillation patients: a systematic review. J Thromb Haemost. 2013;11:975–87. 23441593 10.1111/jth.12177

[4] Sykora D, Firth C, Girardo M, Bhatt S, Matti L, Tseng A, et al. Patient age at diagnosis of peripheral artery disease and its impact on cardiovascular and limb outcomes. Am J Cardiol. 2022;177:144–50. 35760648 10.1016/j.amjcard.2022.04.057

[5] Lip GY, Nieuwlaat R, Pisters R, Lane DA, Crijns HJ. Refining clinical risk stratification for predicting stroke and thromboembolism in atrial fibrillation using a novel risk factor-based approach: the euro heart survey on atrial fibrillation. Chest. 2010;137:263–72. 19762550 10.1378/chest.09-1584

[6] Hindricks G, Potpara T, Dagres N, Arbelo E, Bax JJ, Blomström-Lundqvist C, et al. 2020 ESC Guidelines for the diagnosis and management of atrial fibrillation developed in collaboration with the European Association for Cardio-Thoracic Surgery (EACTS): The Task Force for the diagnosis and management of atrial fibrillation of the European Society of Cardiology (ESC) Developed with the special contribution of the European Heart Rhythm Association (EHRA) of the ESC. Eur Heart J. 2021;42:373–498. 32860505 10.1093/eurheartj/ehaa612

[7] Aboyans V, Ricco JB, Bartelink MEL, Björck M, Brodmann M, Cohnert T, et al. 2017 ESC Guidelines on the Diagnosis and Treatment of Peripheral Arterial Diseases, in collaboration with the European Society for Vascular Surgery (ESVS): document covering atherosclerotic disease of extracranial carotid and vertebral, mesenteric, renal, upper and lower extremity arteriesEndorsed by: the European Stroke Organization (ESO)The task force for the diagnosis and treatment of peripheral arterial diseases of the European Society of Cardiology (ESC) and of the European Society for Vascular Surgery (ESVS). Eur Heart J. 2018;39:763–816. 28886620 10.1093/eurheartj/ehx095

[8] Franchi C, Antoniazzi S, Proietti M, Nobili A, Mannucci PM, Collaborators S-A. Appropriateness of oral anticoagulant therapy prescription and its associated factors in hospitalized older people with atrial fibrillation. Br J Clin Pharmacol. 2018;84:2010–9. 29745441 10.1111/bcp.13631 PMC6089830

[9] Inohara T, Shrader P, Pieper K, Blanco RG, Allen LA, Fonarow GC, et al. Treatment of atrial fibrillation with concomitant coronary or peripheral artery disease: Results from the outcomes registry for better informed treatment of atrial fibrillation II. Am Heart J. 2019;213:81–90. 31129441 10.1016/j.ahj.2019.04.007

[10] Proietti M, Raparelli V, Laroche C, Dan GA, Janion M, Popescu R, et al. Adverse outcomes in patients with atrial fibrillation and peripheral arterial disease: a report from the EURObservational research programme pilot survey on atrial fibrillation. Europace. 2017;19:1439–48. 27940934 10.1093/europace/euw169

[11] Boriani G, Proietti M, Laroche C, Fauchier L, Marin F, Nabauer M, et al. Association between antithrombotic treatment and outcomes at 1-year follow-up in patients with atrial fibrillation: the EORP-AF General Long-Term Registry. Europace. 2019;21:1013–22. 30904925 10.1093/europace/euz032

[12] Tse HF, Teo WS, Siu CW, Chao TF, Park HW, Shimizu W, et al. Prognosis and treatment of atrial fibrillation in Asian cities: 1-year review of the Asia-Pacific Heart Rhythm Society Atrial Fibrillation Registry. Europace : European pacing, arrhythmias, and cardiac electrophysiology : journal of the working groups on cardiac pacing, arrhythmias, and cardiac cellular electrophysiology of the European Society of Cardiology. 2022;24:1889–98. 35025986 10.1093/europace/euab327

[13] Hammwöhner M, Goette A. Ten years of non-vitamin K antagonists oral anticoagulants for stroke prevention in atrial fibrillation: is warfarin obsolete? Eur Heart J Suppl. 2020;22:O28–41. 33380942 10.1093/eurheartj/suaa177 PMC7753780

[14] Hobbs SD, Wilmink AB, Bradbury AW. Ethnicity and peripheral arterial disease. Eur J Vasc Endovasc Surg. 2003;25:505–12. 12787691 10.1053/ejvs.2002.1884

[15] Sebastianski M, Makowsky MJ, Dorgan M, Tsuyuki RT. Paradoxically lower prevalence of peripheral arterial disease in South Asians: a systematic review and meta-analysis. Heart. 2014;100:100–5. 23756656 10.1136/heartjnl-2013-303605

[16] Mandaglio-Collados D, Marín F, Rivera-Caravaca JM. Peripheral artery disease: Update on etiology, pathophysiology, diagnosis and treatment. Med Clin (Barc). 2023;161:344–50. 37517924 10.1016/j.medcli.2023.06.005

[17] Adegbola A, Behrendt CA, Zyriax BC, Windler E, Kreutzburg T. The impact of nutrition on the development and progression of peripheral artery disease: A systematic review. Clin Nutr. 2022;41:49–70. 34864455 10.1016/j.clnu.2021.11.005

[18] Criqui MH, Aboyans V. Epidemiology of peripheral artery disease. Circ Res. 2015;116:1509–26. 25908725 10.1161/CIRCRESAHA.116.303849

[19] Proietti M, Farcomeni A. Association Between Peripheral Artery Disease and Incident Risk of Atrial Fibrillation: Strong Evidence Coming From Population-Based Cohort Studies. J Am Heart Assoc. 2018;7(8):e009126. 29666067 10.1161/JAHA.118.009126 PMC6015415

[20] Cordero A, Alegria E. Sex differences and cardiovascular risk. Heart. 2006;92(2):145–6. 16415182 10.1136/hrt.2005.069187 PMC1860754

[21] Potpara T, Romiti GF, Sohns C. The 2024 European Society of Cardiology Guidelines for Diagnosis and Management of Atrial Fibrillation: A Viewpoint from a Practicing Clinician's Perspective. Thromb Haemost. 2024. 10.1055/a-2434-9244. 10.1055/a-2434-9244 39374908

[22] Shakir A, Khan A, Agarwal S, Clifton S, Reese J, Munir MB, et al. Dual therapy with oral anticoagulation and single antiplatelet agent versus monotherapy with oral anticoagulation alone in patients with atrial fibrillation and stable ischemic heart disease: a systematic review and meta-analysis. J Interv Card Electrophysiol. 2023;66:493–506. 36085242 10.1007/s10840-022-01347-1

[23] Lane DA, Kamphuisen PW, Minini P, Büller HR, Lip GYH. Bleeding risk in patients with atrial fibrillation: the AMADEUS study. Chest. 2011;140:146–55. 21415134 10.1378/chest.10-3270

[24] Gorog DA, Gue YX, Chao TF, Fauchier L, Ferreiro JL, Huber K, et al. Assessment and Mitigation of Bleeding Risk in Atrial Fibrillation and Venous Thromboembolism: Executive Summary of a European and Asia-Pacific Expert Consensus Paper. Thromb Haemost. 2022;122:1625–52. 35793691 10.1055/s-0042-1750385

[25] Mant J, Hobbs FD, Fletcher K, Roalfe A, Fitzmaurice D, Lip GY, et al. Warfarin versus aspirin for stroke prevention in an elderly community population with atrial fibrillation (the Birmingham Atrial Fibrillation Treatment of the Aged Study, BAFTA): a randomised controlled trial. Lancet. 2007;370:493–503. 17693178 10.1016/S0140-6736(07)61233-1

[26] Chao TF, Joung B, Takahashi Y, Lim TW, Choi EK, Chan YH, et al. 2021 Focused update consensus guidelines of the Asia Pacific Heart Rhythm Society on stroke prevention in atrial fibrillation: executive summary. Thromb Haemost. 2022;122:20–47. 34773920 10.1055/s-0041-1739411 PMC8763451

[27] Imberti JF, Mei DA, Vitolo M, Bonini N, Proietti M, Potpara T, et al. Comparing atrial fibrillation guidelines: focus on stroke prevention, bleeding risk assessment and oral anticoagulant recommendations. Eur J Intern Med. 2022;101:1–7. 35525635 10.1016/j.ejim.2022.04.023

[28] Mei DA, Imberti JF, Bonini N, Romiti GF, Corica B, Proietti M, et al. Performance of HAS-BLED and DOAC scores to predict major bleeding events in atrial fibrillation patients treated with direct oral anticoagulants: a report from a prospective European observational registry. Eur J Intern Med. 2024;128:63–70. 38969571 10.1016/j.ejim.2024.06.022

[29] Vitalis A, Shantsila A, Proietti M, Vohra RK, Kay M, Olshansky B, et al. Peripheral arterial disease in patients with atrial fibrillation: the AFFIRM study. Am J Med. 2021;134(4):514–8. 32956630 10.1016/j.amjmed.2020.08.026

[30] Hussain MA, Al-Omran M, Creager MA, Anand SS, Verma S, Bhatt DL. Antithrombotic therapy for peripheral artery disease: recent advances. J Am Coll Cardiol. 2018;71(21):2450–67. 29793635 10.1016/j.jacc.2018.03.483

[31] Levy PJ. Premature lower extremity atherosclerosis: clinical aspects. Am J Med Sci. 2002;323:11–6. 11814136 10.1097/00000441-200201000-00003

[32] Romiti GF, Corica B, Mei DA, Bisson A, Boriani G, Olshansky B, et al. Patterns of comorbidities in patients with atrial fibrillation and impact on management and long-term prognosis: an analysis from the Prospective Global GLORIA-AF Registry. BMC Med. 2024;22:151. 38589864 10.1186/s12916-024-03373-4 PMC11003021

[33] Proietti M, Romiti GF, Corica B, Mei DA, Bonini N, Vitolo M, et al. Features of clinical complexity in European patients with atrial fibrillation: a report from a European Observational Prospective AF Registry. Curr Probl Cardiol. 2023;48: 101752. 37087078 10.1016/j.cpcardiol.2023.101752

[34] Proietti M, Romiti GF, Olshansky B, Lane DA, Lip GYH. Comprehensive management with the ABC (Atrial Fibrillation Better Care) pathway in clinically complex patients with atrial fibrillation: a post hoc ancillary analysis from the AFFIRM trial. J Am Heart Assoc. 2020;9: e014932. 32370588 10.1161/JAHA.119.014932 PMC7660878

[35] Bucci T, Proietti M, Shantsila A, Romiti GF, Teo WS, Park HW, et al. Integrated care for atrial fibrillation using the ABC pathway in the prospective APHRS-AF registry. JACC Asia. 2023;3:580–91. 37614548 10.1016/j.jacasi.2023.04.008 PMC10442886

[36] Romiti GF, Proietti M, Vitolo M, Bonini N, Fawzy AM, Ding WY, et al. Clinical complexity and impact of the ABC (Atrial fibrillation Better Care) pathway in patients with atrial fibrillation: a report from the ESC-EHRA EURObservational Research Programme in AF General Long-Term Registry. BMC Med. 2022;20:326. 36056426 10.1186/s12916-022-02526-7 PMC9440492

[37] Treewaree S, Lip GYH, Krittayaphong R. Non-vitamin K Antagonist Oral Anticoagulant, Warfarin, and ABC Pathway Adherence on Hierarchical Outcomes: Win Ratio Analysis of the COOL-AF Registry. Thromb Haemost. 2024;124(1):69–79. 10.1055/s-0043-1772773. 10.1055/s-0043-1772773 37625457

[38] Corica B, Romiti GF, Mei DA, Proietti M, Zhang H, Guo Y, et al. Efficacy of the ABC pathway for integrated care across phenotypes of patients with atrial fibrillation: a latent-class analysis report from the mAFA-II clinical trial. J Gen Intern Med. 2024. 10.1007/s11606-024-09037-6. 10.1007/s11606-024-09037-6 PMC12045915 39466555

