# Supplementary material for: Peripheral artery disease, antithrombotic treatment and outcomes in European and Asian patients with atrial fibrillation: analysis from two prospective observational registries
Source: BMC Med. 2024 Dec 2;22:567. doi: 10.1186/s12916-024-03792-3 (PMC11610368; doi:10.1186/s12916-024-03792-3)
Supplement: Supplementary file 1 — Additional file 1: Table S1. Ethnic distribution in the two registries. Table S2. Baseline characteristics according to the presence of PAD and Registry of enrolment. Table S3. Pharmacological treatment in the overall population accordingly to PAD. Table S4. Difference between patient included and not in survival analysis. Table S5. Outcome of the study according to PAD. Table S6. Missing data in variables included in the model, stratified by the presence of PAD. Table S7. Incidence rates for the outcomes of the study in patients with PAD, according to Registry of enrolment. Table S8. Subgroup analysis for the risk of primary outcome among subgroups of pharmacological treatment. Figure S1. Subgroup analysis for antithrombotic treatment. Figure S2. Kaplan–Meier curves for the endpoints of the study. Panel A: Composite outcome, Panel B: All-cause Death; Panel C: MACEs; Panel D: Any major bleeding. List of investigators. [file 12916_2024_3792_MOESM1_ESM.docx]

**Peripheral Artery Disease, Antithrombotic Treatment and Outcomes in European and Asian Patients with Atrial Fibrillation:**

**Analysis from Two Prospective Observational Registries**

**Supplementary Materials**

**TABLES**

**Table S1.** Ethnic distribution in the two registries

|  | **European** | **Asian** | **P< 0.001** |
| --- | --- | --- | --- |
| **Caucasian n (%)** | 10042 (92.6) | 21 (0.5) |  |
| **Black, n (%)** | 34 (0.3) | 5 (0.1) |  |
| **Oriental (Chinese, Japanese, etc), n (%)** | 11 (0.1) | 4419 (95.2) |  |
| **South Asian (Indian, Pakistan/Bangladesh), n (%)** | 15 (0.1) | 60 (1.3) |  |
| **Other, n (%)** | 542 (5.0) | 134 (2.9) |  |
| **Unknown, n (%)** | 200 (1.8) | 5 (0.1) |  |

**Table S2.** Baseline characteristics according to the presence of PAD and Registry of enrolment

|  | European No PAD | European PAD | Asian  No Pad | Asian  PAD | P-value |
| --- | --- | --- | --- | --- | --- |
| n | 9970 | 883 | 4586 | 58 |  |
| Age (years) (median [IQR]) | 70.00 [62.00, 77.00] | 74.00 [67.00, 79.00] | 69.00 [61.00, 77.00] | 73.00 [66.25, 83.75] | <0.001 |
| Female, N (%) | 4067 (40.8) | 332 (37.6) | 1571 (34.3) | 16 (27.6) | <0.001 |
| BMI (median [IQR]) | 27.50 [24.80, 31.10] | 27.50 [24.70, 31.20] | 24.70 [22.30, 27.30] | 24.80 [23.30, 26.95] | <0.001 |
| Type of atrial fibrillation, N (%) |  |  |  |  | <0.001 |
| First diagnosed | 1585 (16.2) | 109 (12.5) | 339 (7.4) | 5 (8.6) |  |
| Paroxysmal | 2594 (26.5) | 199 (22.8) | 1924 (42.1) | 19 (32.8) |  |
| Persistent | 1945 (19.9) | 133 (15.3) | 1067 (23.3) | 13 (22.4) |  |
| Long-standing Persistent | 425 (4.3) | 43 (4.9) | 452 (9.9) | 4 (6.9) |  |
| Permanent | 3245 (33.1) | 388 (44.5) | 790 (17.3) | 17 (29.3) |  |
| Cardiovascular Comorbidities |  |  |  |  |  |
| Hypertension, N (%) | 6038 (61.1) | 636 (72.4) | 2758 (60.5) | 43 (74.1) | <0.001 |
| Diabetes, N (%) | 2153 (21.7) | 317 (36.1) | 1089 (24.1) | 26 (44.8) | <0.001 |
| Dyslipidemia, N (%) | 3778 (39.5) | 510 (58.9) | 1681 (37.2) | 35 (61.4) | <0.001 |
| Coronary artery disease, N (%) | 2551 (27.1) | 410 (49.3) | 861 (19.0) | 21 (38.9) | <0.001 |
| Heart failure, N (%) | 3680 (37.2) | 515 (58.9) | 950 (20.9) | 20 (35.1) | <0.001 |
| LVEF (%) (median [IQR]) | 55.00 [45.00, 62.00] | 54.00 [42.00, 60.00] | 60.00 [55.00, 65.00] | 60.00 [50.00, 68.50] | <0.001 |
| Previous thromboembolic events, N (%) | 1032 (10.4) | 200 (22.9) | 494 (10.9) | 17 (29.3) | <0.001 |
| Other Comorbidities |  |  |  |  |  |
| Chronic kidney disease, N (%) | 1103 (11.1) | 234 (26.9) | 333 (7.3) | 20 (34.5) | <0.001 |
| Malignancy (current or prior), N (%) | 685 (6.9) | 117 (13.4) | 361 (7.9) | 9 (15.5) | <0.001 |
| Previous hemorrhagic events, N (%) | 507 (5.1) | 58 (6.7) | 347 (7.6) | 6 (10.3) | <0.001 |
| Anemia, N (%) | 468 (4.7) | 110 (12.5) | 321 (7.0) | 12 (20.7) | <0.001 |
| CHA2DS2-VASc (median [IQR]) | 3.00 [2.00, 4.00] | 5.00 [4.00, 6.00] | 3.00 [1.00, 4.00] | 5.00 [3.00, 6.00] | <0.001 |
| HAS-BLED (median [IQR]) | 1.00 [1.00, 2.00] | 2.00 [1.00, 3.00] | 1.00 [1.00, 2.00] | 2.00 [1.00, 3.00] | <0.001 |
| EHRA score III-IV, N (%) | 1928 (19.3) | 177 (20.1) | 279 (6.1) | 1 (1.7) | <0.001 |

**Table S3.** Pharmacological treatment in the overall population accordingly to PAD

|  | **No PAD** | **PAD** | **P** |
| --- | --- | --- | --- |
| **Type of antithrombotic treatment** |  |  | **<0.001** |
| None |  |  |  |
| APT | 1159/14545 (8.0) | 52/941 (5.5) |  |
| OAC | 1122/14545 (7.7) | 113/941 (12.0) |  |
| OAC+APT | 10851/14545 (74.6) | 564/941 (59.9) |  |
| **ACE-inhibitors, n (%)** | 1413/14545 (9.7) | 212/941 (22.5) | **<0.001** |
| **ARBs, n (%)** | 4783/14499 (33.0) | 452/939 (48.1) | 0.357 |
| **Beta-blockers, n (%)** | 3060/14498 (21.1) | 186/940 (19.8) | **<0.001** |
| **Aldosterone blocker, n (%)** | 9142/14496 (63.1) | 666/940 (70.9) | **<0.001** |
| **Diuretics, n (%)** | 2045/14498 (14.1) | 232/940 (24.7) | **<0.001** |
| **Digoxin, n (%)** | 5967/14498 (41.2) | 609/939 (64.9) | **<0.001** |
| **Calcium channel blockers, n (%)** | 1914/14500 (13.2) | 163/940 (17.3) | 0.111 |
| **Non-DHP CCB, n (%)** | 2666/14498 (18.4) | 193/940 (20.5) | 0.169 |
| **Statin, n (%)** | 1145/14504 (7.9) | 62/940 (6.6) | **<0.001** |
| **Oral antidiabetics, n (%)** | 5743/14493 (39.6) | 545/939 (58.0) | **<0.001** |
| **Insulin, n (%)** | 2158/14497 (14.9) | 212/940 (22.6) | **<0.001** |
| **Any rate control** | 602/14499 (4.2) | 117/940 (12.4) | **<0.001** |
| **IC or III class antiarrhythmic drugs** | 10334/14503 (71.3) | 751/940 (79.9) | **0.005** |

Legend: APT, antiplatelet; ARB, angiotensin receptor blocker; CCB, calcium channel blocker; NOAC, non-vitamin K antagonist; non-DHP, non-dihydropyridine; OAC, oral anticoagulant

**Table S4.** Difference between patient included and not in survival analysis

|  | Included  13606 (88%) | Not Included  1891 (12%) | p |
| --- | --- | --- | --- |
| **Age (years) (median [IQR])** | 70.00 [62.00, 77.00] | 69.00 [61.00, 77.00] | 0.003 |
| **Female, N (%)** | 5246 (38.6) | 740 (39.1) | 0.648 |
| **BMI (median [IQR])** | 26.70 [24.00, 30.10] | 26.10 [23.70, 29.30] | <0.001 |
| **Type of atrial fibrillation, N (%)** |  |  | 0.155 |
| First diagnosed | 1794 (13.4) | 244 (13.0) |  |
| Paroxysmal | 4114 (30.7) | 622 (33.1) |  |
| Persistent | 2801 (20.9) | 357 (19.0) |  |
| Long-standing Persistent | 805 (6.0) | 119 (6.3) |  |
| Permanent | 3904 (29.1) | 536 (28.5) |  |
| **Cardiovascular Disease** |  |  |  |
| Hypertension, N (%) | 8297 (61.5) | 1178 (62.8) | 0.287 |
| Diabetes, N (%) | 3168 (23.5) | 417 (22.2) | 0.234 |
| Dyslipidemia, N (%) | 5311 (40.4) | 693 (37.5) | 0.021 |
| Coronary artery disease, N (%) | 3362 (25.8) | 481 (27.2) | 0.207 |
| Heart failure, N (%) | 4534 (33.6) | 631 (33.6) | 1.000 |
| LVEF (%) (median [IQR]) | 58.00 [50.00, 63.00] | 57.00 [47.00, 62.00] | 0.003 |
| Previous thromboembolic events, N (%) | 1550 (11.5) | 193 (10.3) | 0.125 |
| **Comorbidities** |  |  |  |
| Chronic kidney disease, N (%) | 1488 (11.0) | 202 (10.7) | 0.732 |
| CrCl CG (median [IQR]) | 71.59 [52.75, 93.16] | 70.27 [51.77, 91.26] | 0.174 |
| Malignancy (current or prior), N (%) | 1062 (7.8) | 110 (5.8) | 0.003 |
| Previous hemorrhagic events, N (%) | 812 (6.0) | 106 (5.6) | 0.549 |
| Anemia, N (%) | 806 (5.9) | 105 (5.6) | 0.569 |
| **CHA2DS2-VASc (median [IQR])** | 3.00 [2.00, 4.00] | 3.00 [2.00, 4.00] | 0.080 |
| **HAS-BLED (median [IQR])** | 1.00 [1.00, 2.00] | 1.00 [1.00, 2.00] | 0.796 |
| **EHRA class high (III-IV), N (%)** | 2110 (15.5) | 275 (14.6) | 0.295 |
| **Peripheral artery disease, N (%)** | 831 (6.1) | 110 (5.8) | 0.657 |

Legend: CG, Cockcroft-Gault; CKD, chronic kidney disease; CrCl, creatinine clearance; IQR, interquartile range; LVEF, left ventricular ejection fraction; TE, thromboembolic events.

**Table S5.** Outcome of the study according to PAD

|  | **No PAD** | **PAD** | **P** |
| --- | --- | --- | --- |
| **Primary Endpoint** |  |  |  |
| Composite Outcome, n (%) | 1448 (11.3) | 216 (26.0) | **<0.001** |
| **Secondary Endpoint** |  |  |  |
| All-cause death, n (%) | 935 (6.9) | 149 (17.2) | **<0.001** |
| MACEs, n (%) | 894 (6.8) | 138 (15.8) | **<0.001** |
| Any major bleeding, n (%) | 233 (1.7) | 25 (2.9) | **0.021** |

Legend MACE, major adverse cardiovascular events.

**Table S6.** Missing data in variables included in the model, stratified by the presence of PAD

|  | **No PAD** | **PAD** |  |
| --- | --- | --- | --- |
| n | 14556 | 941 |  |
| **Composite outcome, N (%)** | 1781/14556 (12.2) | 110/941 (11.7) |  |
| **Age, N (%)** | 7/14556 (0.0) | 0/941 (0.0) |  |
| **Sex, N (%)** | 0/14556 (0.0) | 0/941 (0.0) |  |
| **Type of atrial fibrillation, N (%)** | 190/14556 (1.3) | 11/941 (1.2) |  |
| **Ehra class, N (%)** | 0/14556 (0.0) | 1/941 (0.1) |  |
| **Coronary artery disease, N (%)** | 619/14556 (4.3) | 56/941 (6.0) |  |
| **Previous Thromboembolic events, N (%)** | 121/14556 (0.8) | 9/941 (1.0) |  |
| **Diabetes, N (%)** | 126/14556 (0.9) | 5/941 (0.5) |  |
| **Hypertension, N (%)** | 115/14556 (0.8) | 4/941 (0.4) |  |
| **Heart failure, N (%)** | 130/14556 (0.9) | 10/941 (1.1) |  |
| **Oral anticoagulant therapy, N (%)** | 6/14556 (0.0) | 0/941 (0.0) |  |
| **Statin therapy, N (%)** | 63/14556 (0.4) | 2/941 (0.2) |  |

**Table S7.** Incidence rates for the outcomes of the study in patients with PAD, according to Registry of enrolment

|  | **European** | | **Asian** | |
| --- | --- | --- | --- | --- |
| **Primary Endpoint** | N (%) | IR/100 p-years  [95% CI] | N (%) | IR/100 p-years [95% CI] |
| Composite Outcome | 211 (27.0) | 16.27 [14.14-18.62] | 5 (10.2) | 11.02 [3.58-25.71] |
| **Secondary Endpoint** |  |  |  |  |
| All-cause death | 144 (17.7) | 10.30 [8.69-12.13] | 5 (9.6) | 10.25 [3.33-23.92] |
| MACEs | 137 (16.6) | 10.56 [8.87-12.49] | 1 (2.0) | 2.20 [0.06-12.28] |
| Any major bleeding | 23 (2.9) | 1.68 [1.06-2.52] | 2 (4.1) | 4.70 [0.57-16.98] |

Legend CI, confidence interval IR, incidence rate; MACE, major adverse cardiovascular events; p-years, person years.

**Table S8.** Subgroup analysis for the risk of primary outcome among subgroups of pharmacological treatment.

|  | **HR [95% CI]** | **Pint** |
| --- | --- | --- |
| **OAC therapy** |  |  |
| No  Yes | 1.51 [1.08-2.12]  1.22 [1.00-1.48] | **0.272** |
| **Statin therapy** |  |  |
| No  Yes | 1.85 [1.47-2.32]  0.92 [0.72-1.18] | **<0.001** |

**Legend.** HR, hazard ratio; OAC, oral anticoagulant; Pint, p for interaction.

**FIGURES**

**Figure S1.** Subgroup analysis for antithrombotic treatment


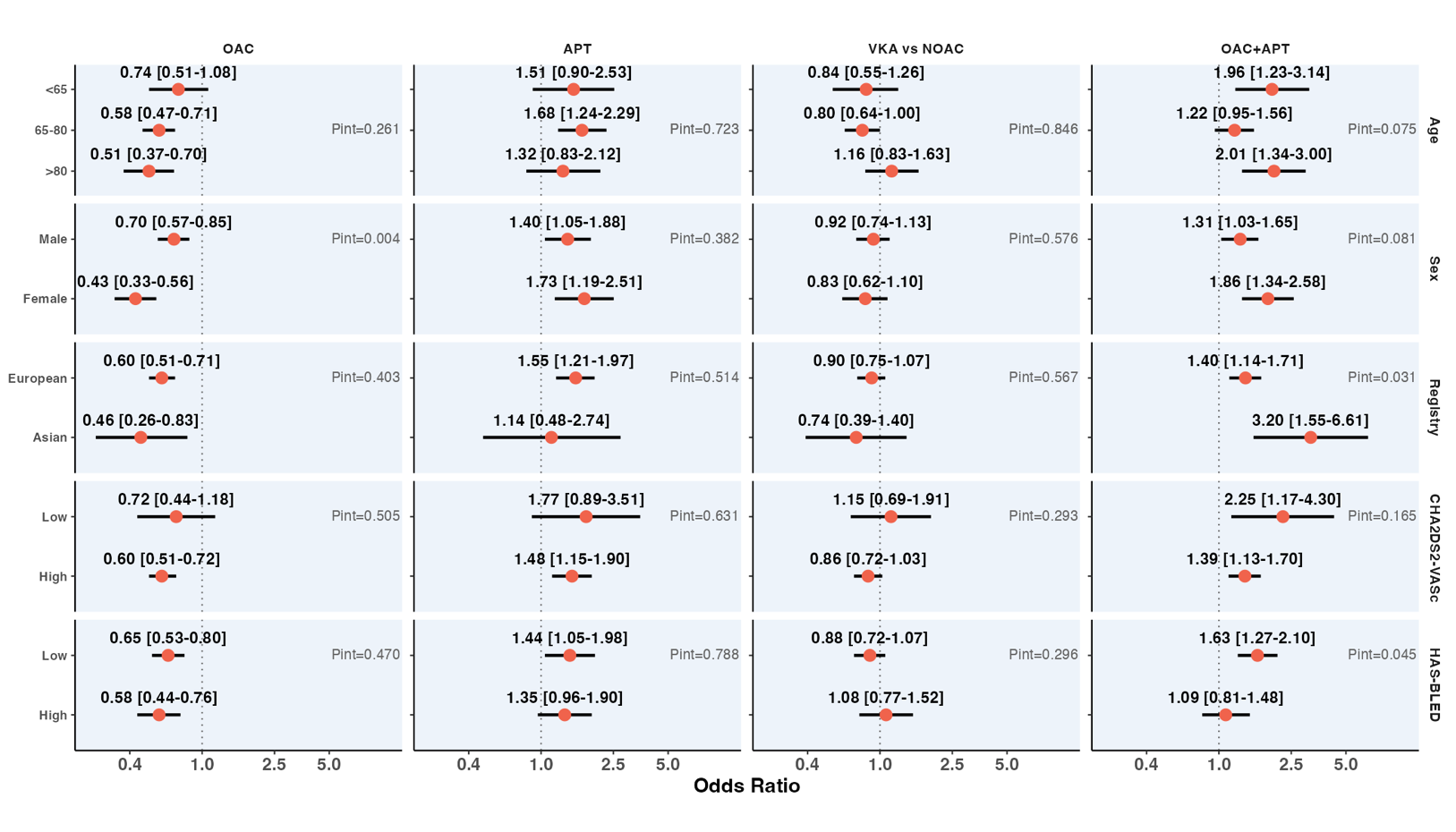


Legend. APT, antiplatelet; Non Vitamin K oral anticoagulant; OAC, oral anticoagulant; VKA, vitamin-K antagonist

**Figure S2.** Kaplan-Meier curves for the endpoints of the study.

Panel A: Composite outcome, Panel B: All-cause Death; Panel C: MACEs; Panel D: Any major bleeding.


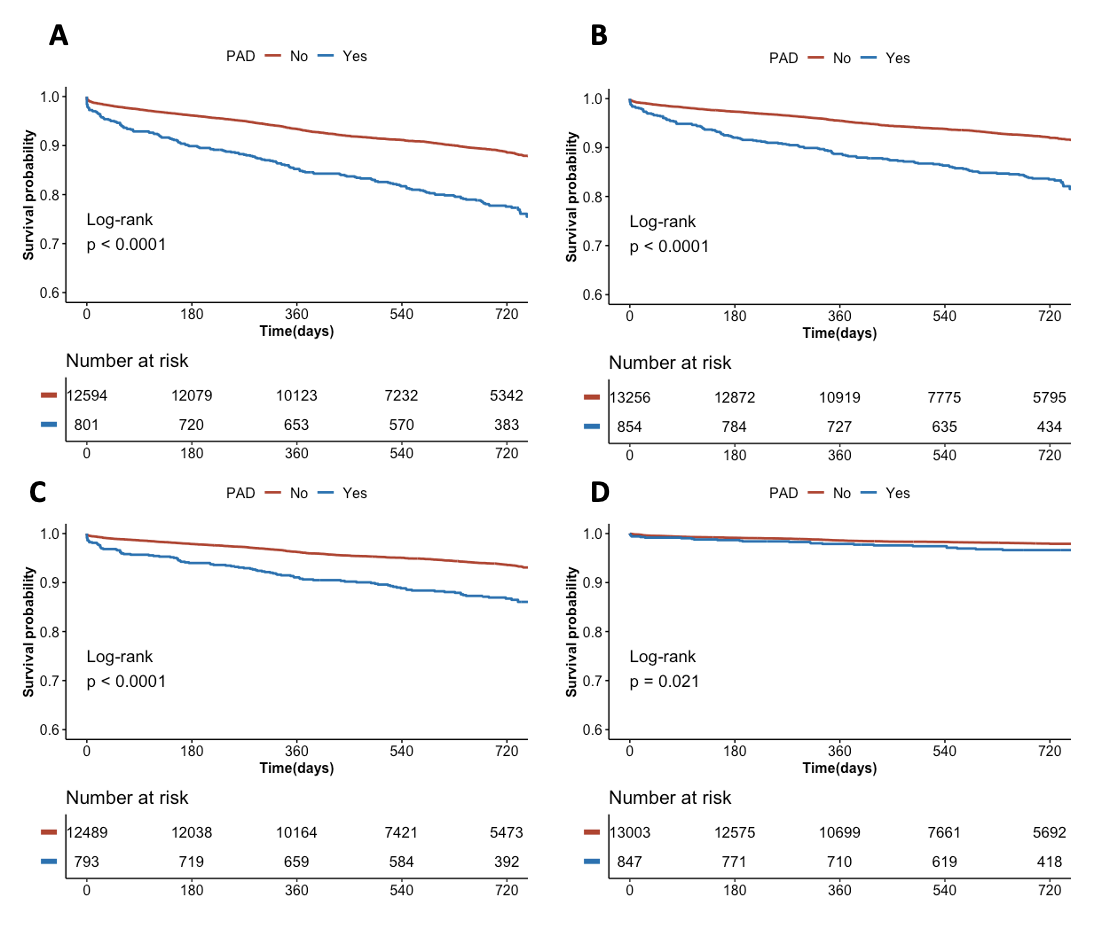


**List of investigators**

*EURObservational Research Programme Atrial Fibrillation Long-Term General Registry Investigators
Executive committee*: G. Boriani (Chair), G.Y.H. Lip, L. Tavazzi, A. P. Maggioni,
G-A. Dan, T. Potpara, M. Nabauer, F. Marin, Z. Kalarus, L. Fauchier, R. Ferrari, A. Shantsila.

*Steering Committee (National Coordinators)*: A. Goda, *University Hospital Center ‘Mother Tereza’, Tirana, Albania*; G. Mairesse, *Cliniques du Sud-Luxembourg, Arlon, Belgium;* T. Shalganov, *National Heart Hospital, Sofia, Bulgaria;* L. Antonia-
des, *Nicosia General Hospital, Latsia, Cyprus;* M. Taborsky, *University Hospital Olomouc, Olomouc, Czech Republic;* S. Riahi, *Aalborg University Hospital, Aalborg, Denmark;* P. Muda, *University of Tartu, Tartu, Estonia;* I. García Bolao, *Navarra Institute for Health Research, Pamplona, Spain;* O. Piot, *Centre Cardiologique du Nord, Saint-Denis, France; M. Nabauer, Ludwig-Maximilians-University, Munich, Germany; K. Etsadashvili, G. Chapidze Emergency Cardiology Center, Tbilisi, Georgia; EN. Simantirakis, University Hospital of Heraklion, School of Medicine, University of Crete, Heraklion, Crete, Greece; M. Haim, Soroka Medical Center, Beer Sheva, Israel; A. Azhari, J. Najafian, Cardiovascular Research Institute, Isfahan University of Medical Sciences, Isfahan, Iran; M. Santini, San Filippo Neri Hospital, Rome, Italy; E. Mirrakhimov, National Center of Cardiology and Internal Medicine, Bishkek, Kyrgyzstan; K. Kulzida, Scientific-Research Institute of Cardiology and Internal Diseases, Almaty, Republic of Kazakhstan; A. Erglis, Pauls Stradins Clinical University Hospital University of Latvia Riga Latvia; L. Poposka, University Clinic of Cardiology, Faculty of Medicine, Ss Cyril and Methodius University of Skopje, Skopje, Republic of Macedonia; MR. Burg, Mater Dei Hospital, Triq Dun Karm Psaila, Malta; H. Crijns, Ö. Erküner, Cardiovascular Research Institute Maastricht (CARIM), Maastricht University Medical Centre, Maastricht, The Netherlands; D. Atar, Oslo University Hospital Ullevål and Institute of Clinical Sciences, University of Oslo, Oslo, Norway; R. Lenarczyk, Silesian Center for Heart Disease, Zabrze, Poland; M. Martins Oliveira, Hospital Santa Marta, Lisbon, Portugal; D. Shah, Department of Medicine Specialities, University Hospital Geneva, Geneva, Switzerland; G-A. Dan, Colentina University Hospital, Bucharest, Romania; E. Serdechnaya, Northern State Medical University, Arkhangelsk, Russia; T. Potpara, Cardiology Clinic, Clinical Center of Serbia, Belgrade, Serbia; E. Diker, Başakşehir Çam and Sakura City Hos- pital, Istanbul, Turkey; G.Y.H. Lip, D. Lane; City Hospital, University of Birmingham, Birmingham, United Kingdom.*

*Investigators: Albania—Durrës: E. Zëra; Tirana: U. Ekmekçiu, V. Paparisto, M. Tase; Tirana: H. Gjergo, J. Dragoti, A. Goda. Belgium—Bastogne: M. Ciutea, N. Ahadi, Z. el Husseini, M. Raepers; Gilly: J. Leroy, P. Haushan, A. Jourdan; Haine Saint Paul: C. Lepiece; Hasselt: L. Desteghe, J. Vijgen, P. Koopman, G. Van Genechten, H. Heidbuchel; Kortrijk: T. Boussy, M. De Coninck, H. Van Eeckhoutte, N. Bouckaert; La Louviere: A. Friart, J. Boreux, C. Arend; Liege: P. Evrard; Liège: L. Stefan, E. Hoffer, J. Herzet, M. Massoz; Liège: C. Celentano, M. Sprynger, L. Pierard; Liège: P. Melon; Overpelt: B. Van Hauwaert, C. Kuppens, D. Faes, D. Van Lier, A. Van Dorpe; Waremme: A. Gerardy; Yvoir: O. Deceuninck, O. Xhaet, F. Dormal, E. Ballant, D. Blommaert. Bulgaria—Pleven: D. Yakova, M. Hristov, T. Yncheva, N. Stancheva, S. Tisheva; Plovdiv: M. Tokmakova, F. Nikolov, D. Gencheva; Sofia: T. Shalganov, B. Kunev, M. Stoyanov; Sofia: D. Marchov, V. Gelev, V. Traykov; Varna: A. Kisheva, H. Tsvyatkov, R. Shtereva, S. Bakalska-Geor- gieva, S. Slavcheva, Y. Yotov. Czech Republic—Ústí nad Labem: M. Kubíčková. Denmark—Aalborg: A. Marni Joensen, A. Gammelmark, L. Hvilsted Rasmussen, P. Dinesen, S. Riahi, S. Krogh Venø, B. Sorensen, A. Korsgaard, K. Andersen, C. Fragtrup Hellum; Esbjerg: A. Svenningsen, O. Nyvad, P. Wiggers; Herning: O. May, A. Aarup, B. Graversen, L. Jensen, M. Andersen, M. Svejgaard, S. Vester, S. Hansen, V. Lynggaard. Estonia— Tallinn: R. Vettus; Tartu: P. Muda. Elche, Alicante: A. Maestre; Toledo: S. Castaño. France—Abbeville: S. Cheggour; Abbeville: J. Poulard, V. Mouquet, S. Leparrée; Aix-en-Provence: J. Bouet, J. Taieb; Amiens: A. Doucy, H. Duquenne; Angers: A. Furber, J. Dupuis, J. Rautureau; Aurillac: M. Font, P. Damiano; Avignon Cedex: M. Lacrimini; Brest: J. Abalea, S. Boismal, T. Menez, J. Mansourati; Chartres: G. Range, H. Gorka, C. Laure, C. Vassalière; Creteil: N. Elbaz, N. Lellouche, K. Djouadi; Montpellier: F. Roubille, D. Dietz, J. Davy; Nimes: M. Granier, P. Winum, C. Leperchois-Jacquey; Paris: H. Kassim, E. Marijon, J. Le Heuzey; Paris: J. Fedida, C. Maupain, C. Himbert, E. Gandjbakhch, F. Hidden-Lucet, G. Duthoit, N. Badenco, T. Chastre, X. Waintraub, M. Oudihat, J. Lacoste, C. Stephan; Pau: H. Bader, N. Delarche, L. Giry; Pessac: D. Arnaud, C. Lopez, F. Boury, I. Brunello, M. Lefèvre, R. Mingam, M. Haissaguerre; Rennes: M. Le Bidan, D. Pavin, V. Le Moal, C. Leclercq; Saint Denis: O. Piot, T. Beitar; Saint Etienne: I. Martel, A. Schmid, N. Sadki, C. Romeyer- Bouchard, A. Da Costa; Tours: I. Arnault, M. Boyer, C. Piat, L. Fauchier. FYR Macedonia—Bitola: N. Lozance, S. Nastevska; Ohrid: A. Doneva, B. Fortoma- roska Milevska, B. Sheshoski, K. Petroska, N. Taneska, N. Bakrecheski; Skopje: K. Lazarovska, S. Jovevska, V. Ristovski, A. Antovski; Skopje: E. Lazarova, I. Kotlar, J. Taleski, L. Poposka, S. Kedev; Skopje: N. Zlatanovik; Štip: S. Jordanova, T. Bajraktarova Proseva, S. Doncovska. Georgia—Tbilisi: D. Maisuradze, A. Esakia, E. Sagirashvili, K. Lartsuliani, N. Natelashvili, N. Gumberidze, R. Gvenetadze; Tbilisi: K. Etsadashvili, N. Gotonelia, N. Kuridze; Tbilisi: G. Papiashvili, I. Menabde. Germany—Aachen: S. Glöggler, A. Napp, C. Lebherz, H. Romero, K. Schmitz, M. Berger, M. Zink, S. Köster, J. Sachse, E. Vonderhagen, G. Soiron, K. Mischke; Bad Reichenhall: R. Reith, M. Schneider; Berlin: W. Rieker; Biberach: D. Boscher, A. Taschareck, A. Beer; Boppard: D. Oster; Brandenburg: O. Ritter, J. Adamczewski, S. Walter; Chemnitz: A. Frommhold, E. Luckner, J. Richter, M. Schellner, S. Landgraf, S. Bartholome; Chemnitz: R. Naumann, J. Schoeler; Dachau: D. Westermeier, F. William, K. Wilhelm, M. Maerkl; Detmold: R. Oekinghaus, M. Denart, M. Kriete, U. Tebbe; Ebersbach: T. Scheibner; Erlangen: M. Gruber, A.*

*Gerlach, C. Beckendorf, L. Anneken, M. Arnold, S. Lengerer, Z. Bal, C. Uecker, H. Förtsch, S. Fechner, V. Mages; Friedberg: E. Martens, H. Methe; Göttingen: T. Schmidt; Hamburg: B. Schaeffer, B. Hoffmann, J. Moser, K. Heitmann, S. Willems, S. Willems; Hartmannsdorf: C. Klaus, I. Lange; Heidelberg: M. Durak, E. Esen; Itzehoe: F. Mibach, H. Mibach; Kassel: A. Utech; Kirchzarten: M. Gabelmann, R. Stumm, V. Ländle; Koblenz: C. Gartner, C. Goerg, N. Kaul, S. Messer, D. Burkhardt, C. Sander, R. Orthen, S. Kaes; Köln: A. Baumer, F. Dodos; Königsbrück: A. Barth, G. Schaeffer; Leisnig: J. Gaertner, J. Winkler; Leverkusen: A. Fahrig, J. Aring, I. Wenzel; Limburg: S. Steiner, A. Kliesch, E. Kratz, K. Winter, P. Schneider; Ludwigsburg: A. Haag, I. Mutscher, R. Bosch; Markkleeberg: J. Taggeselle, S. Meixner; Meissen: A. Schnabel; Meppen: A. Shamalla, H. Hötz, A. Korinth; Merzig: C. Rheinert; Moosburg: G. Mehltretter; Mühldorf: B. Schön, N. Schön, A. Starflinger, E. Englmann; Munich: G. Baytok, T. Laschinger, G. Ritscher; Munich: A. Gerth; Münster: D. Dechering, L. Eckardt; Nienburg: M. Kuhlmann, N. Proskynitopoulos; Paderborn: J. Brunn, K. Foth; Pirna: C. Axthelm, H. Hohensee, K. Eberhard, S. Turbanisch; Plauen: N. Hassler, A. Koestler; Riesa: G. Stenzel; Riesa: D. Kschiwan, M. Schwefer, S. Neiner, S. Hettwer; Rotenburg a.d. Fulda: M. Haeussler-Schuchardt, R. Degenhardt, S. Sennhenn, S. Steiner; Starnberg: M. Brendel; Westerstede: A. Stoehr, W. Widjaja, S. Loehndorf, A. Logemann, J. Hoskamp, J. Grundt; Zorneding: M. Block; Zwiesel: R. Ulrych, A. Reithmeier, V. Panagopoulos. Italy—Bologna: C. Martignani, D. Bernucci, E. Fantecchi, I. Diemberger, M. Ziacchi, M. Biffi, P. Cimaglia, J. Frisoni, G. Boriani; Firenze: I. Giannini, S. Boni, S. Fumagalli, S. Pupo, A. Di Chiara, P. Mirone; Modena: E. Fantecchi, G. Boriani, F. Pesce, C. Zoccali, V.L. Malavasi. Kazakhstan—Almaty: A. Mussagaliyeva, B. Ahyt, Z. Salihova, K. Koshum-Bayeva. Kyrgyzstan—Bishkek: A. Kerimkulova, A. Bairamukova, E. Mirrakhimov. Latvia—Riga: B. Lurina, R. Zuzans, S. Jegere, I. Mintale, K. Kupics, K. Jubele, A. Erglis, O. Kalejs. Malta—Birkirkara: K. Vanhear, M. Burg, M. Cachia, E. Abela, S. Warwicker, T. Tabone, R. Xuereb. Montenegro—Podgorica: D. Asanovic, D. Drakalovic, M. Vukmirovic, N. Pavlovic, L. Music, N. Bulatovic, A. Boskovic. Netherlands—Almere: H. Uiterwaal, N. Bijsterveld; Amsterdam: J. De Groot, J. Neefs, N. van den Berg, F. Piersma, A. Wilde; Delfzijl: V. Hagens; Enschede: J. Van Es, J. Van Opstal, B. Van Rennes, H. Verheij, W. Breukers; Heerenveen: G. Tjeerdsma, R. Nijmeijer, D. Wegink, R. Binnema; Hengelo: S. Said; Maastricht: Ö. Erküner, S. Philippens, W. van Doorn, H. Crijns; Rotterdam: T. Szili-Torok, R. Bhagwandien, P. Janse, A. Muskens; s-Hertogenbosch: M. van Eck, R. Gevers, N. van der Ven; Venlo: A. Duygun, B. Rahel, J. Meeder. Norway—Oslo: A. Vold, C. Holst Hansen, I. Engset, D. Atar. Poland—Bytom: B. Dyduch-Fejklowicz, E. Koba, M. Cichocka; Cieszyn: A. Sokal, A. Kubicius, E. Pruchniewicz; Gliwice: A. Kowalik-Sztylc, W. Czapla; Katowice: I. Mróz, M. Kozlowski, T. Pawlowski, M. Tendera; Katowice: A. Winiarska-Filipek, A. Fidyk, A. Slowikowski, M. Haberka, M. Lachor-Broda, M. Biedron, Z. Gasior; Kielce: M. Kołodziej, M. Janion; Kielce: I. Gorczyca-Michta, B. Wozakowska- Kaplon; Łódź: M. Stasiak, P. Jakubowski, T. Ciurus, J. Drozdz; Łódź: M. Simiera, P. Zajac, T. Wcislo, P. Zycinski, J. Kasprzak; Nysa: A. Olejnik, E. Harc-Dyl, J. Miarka, M. Pasieka, M. Ziemińska-Łuć, W. Bujak; Opoczno: A. Śliwiński, A. Grech, J. Morka, K. Petrykowska, M. Prasał; Opole: G. Hordyński, P. Feusette, P. Lipski, A. Wester; Radlin: W. Streb; Rzeszów: J. Romanek, P. Woźniak, M. Chlebuś, P. Szafarz, W. Stanik; Szczecin: M. Zakrzewski, J. Kaźmierczak; Szczecin: A. Przybylska, E. Skorek, H. Błaszczyk, M. Stępień, S. Szabowski, W. Krysiak, M. Szymańska; Tarnów: J. Karasiński, J. Blicharz, M. Skura; Warsaw: K. Hałas, L. Michalczyk, Z. Orski, K. Krzyżanowski, A. Skrobowski; Warsaw: L. Zieliński, M. Tomaszewska- Kiecana, M. Dłużniewski; Warsaw: M. Kiliszek, M. Peller, M. Budnik, P. Balsam, G. Opolski, A. Tymińska, K. Ozierański, A. Wancerz; Warsaw: A. Borowiec, E. Majos, R. Dabrowski, H. Szwed; Zabrze: A. Musialik-Lydka; Zabrze: A. Leopold-Jadczyk, E. Jedrzejczyk-Patej, M. Koziel, R. Lenarczyk, M. Mazurek, Z. Kalarus; Zabrze: K. Krzemien-Wolska, P. Starosta, E. Nowalany-Kozielska; Zakopane: A. Orzechowska, M. Szpot, M. Staszel. Portugal—Almada: S. Almeida, H. Pereira, L. Brandão Alves, R. Miranda, L. Ribeiro; Carnaxide Lisboa: F. Costa, F. Morgado, P. Carmo, P. Galvao Santos, R. Bernardo, P. Adragão; Santarém: G. Ferreira da Silva, M. Peres, M. Alves, M. Leal; Vila Real: A. Cordeiro, P. Magalhães, P. Fontes, S. Leão; Viseu: A. Delgado, A. Costa, B. Marmelo, B. Rodrigues, D. Moreira, J. Santos, L. Santos. Romania—Arad: A. Terchet, D. Darabantiu, S. Mercea, V. Turcin Halka, A. Pop Moldovan; Brasov: A. Gabor, B. Doka, G. Catanescu, H. Rus, L. Oboroceanu, E. Bobescu; Bucharest: R. Popescu, A. Dan, A. Buzea, I. Daha, G. Dan, I. Neuhoff; Bucharest: M. Baluta, R. Ploesteanu, N. Dumitrache, M. Vintila; Bucharest: A. Daraban, C. Japie, E. Badila, H. Tewelde, M. Hostiuc, S. Frunza, E. Tintea, D. Bartos; Bucharest: A. Ciobanu, I. Popescu, N. Toma, C. Gherghinescu, D. Cretu, N. Patrascu, C. Stoicescu, C. Udroiu, G. Bicescu, V. Vintila, D. Vinereanu, M. Cinteza, R. Rimbas; Iași: M. Grecu; Oradea: A. Cozma, F. Boros, M. Ille, O. Tica, R. Tor, A. Corina, A. Jeewooth, B. Maria, C. Georgiana, C. Natalia, D. Alin, D. Dinu-Andrei, M. Livia, R. Daniela, R. Larisa, S. Umaar, T. Tamara, M. Ioachim. Popescu; Târgu Mureș: D. Nistor, I. Sus, O. Coborosanu; Timișoara: N. Alina-Ramona, R. Dan, L. Petrescu; Timișoara: G. Ionescu, I. Popescu, C. Vacarescu, E. Goanta, M. Mangea, A. Ionac, C. Mornos, D. Cozma, S. Pescariu. Russian Federation—Arkhangelsk: E. Solodovnicova, I. Soldatova, J. Shutova, L. Tjuleneva, T. Zubova, V. Uskov; Arkhangelsk: D. Obukhov, G. Rusanova; Arkhangelsk: I. Soldatova, N. Isakova, S. Odinsova, T. Arhipova; Arkhangelsk: E. Kazakevich, E. Serdechnaya, O. Zavyalova; Saint-Petersburg: T. Novikova; Saint-Petersburg: I. Riabaia, S. Zhigalov; Saint-Petersburg: E. Drozdova, I. Luchkina, Y. Monogarova; Vladivostok: D. Hegya, L. Rodionova, L. Rodionova, V. Nevzorova; Vladivostok: I. Soldatova, O. Lusanova. Serbia—Belgrade: A. Arandjelovic, D. Toncev, M. Milanov, N. Sekularac; Belgrade: M. Zdravkovic, S. Hinic, S. Dimkovic, T. Acimovic, J. Saric; Belgrade: M. Polovina, T. Potpara, B. Vujisic-Tesic, M. Nedeljkovic; Belgrade: M. Zlatar, M. Asanin; Belgrade: V. Vasic, Z. Popovic; Belgrade: D. Djikic, M. Sipic, V. Peric, B. Dejanovic, N. Milosevic; Belgrade: A. Stevanovic, A. Andric, B. Pencic, M. Pavlovic-Kleut, V. Celic; Kragujevac: M. Pavlovic, M. Petrovic, M. Vuleta, N. Petrovic, S. Simovic, Z. Savovic, S. Milanov, G. Davidovic, V. Iric-Cupic; Niška Banja: D. Simonovic, M. Stojanovic, S. Stojanovic, V. Mitic, V. Ilic, D. Petrovic, M. Deljanin Ilic, S. Ilic, V. Stoickov; Pirot: S. Markovic; Šabac: S. Kovacevic. Spain—Alicante: A. García Fernandez; Benalmadena: A. Perez Cabeza; Córdoba: M. Anguita; Elche, Alicante: A. Maestre; Toledo: S. Castaño Granada: L. Tercedor Sanchez; Huarte: E. Mau, J. Loayssa, M. Ayarra, M. Carpintero; Madrid: I. Roldán Rabadan; Murcia: M. Leal; Murcia: M. Gil Ortega; Murcia: A. Tello Montoliu, E. Orenes Piñero, S. Manzano Fernández, F. Marín, A. Romero Aniorte, A. Veliz Martínez, M. Quintana Giner; Madrid: M. Ciudad; Pamplona: G. Ballesteros, M. Palacio, O. Alcalde, I. García-Bolao; San Juan de Alicante: V. Bertomeu Gonzalez; Santiago de Compostela: F. Otero-Raviña, J. García Seara, J. Gonzalez Juanatey. Switzerland—Geneva: N. Dayal, P. Maziarski, P. Gentil-Baron, D. Shah. Turkey—Adana: M. Koç; Afyon: E. Onrat, I. E. Dural; Ankara: K. Yilmaz, B. Özin; Ankara: S. Tan Kurklu, Y. Atmaca; Ankara: U. Canpolat, L. Tokgozoglu; Ankara: A. K. Dolu, B. Demirtas, D. Sahin; Ankara: O. Ozcan Celebi, E. Diker; Antalya: G. Gagirci; Bayraklı/Izmir: U.O.Turk; Bursa: H. Ari; Diyarbakır: N. Polat, N. Toprak; Gaziantep: M. Sucu; Görükle-Bursa: O. Akin Serdar; Istanbul: A. Taha Alper; Istanbul: A. Kepez; Istanbul: Y. Yuksel; Kurupelit - Samsun: A. Uzunselvi, S. Yuksel, M. Sahin; Merkez/Düzce: O. Kayapinar; Mersin: T. Ozcan; Sivas: H. Kaya, M. B. Yilmaz; Trabzon: M. Kutlu; Yüreğir-Adana: M. Demir. UK—Barnstaple: C. Gibbs, S. Kaminskiene, M. Bryce, A. Skinner, G. Belcher, J. Hunt, L. Stancombe, B. Holbrook, C. Peters, S. Tettersell; Birmingham: A. Shantsila, D. Lane, K. Senoo, M. Proietti, K. Russell, P. Domingos, S. Hussain, J. Partridge, R. Haynes, S. Bahadur, R. Brown, S. McMahon, G. Y H Lip; Blackburn: J. McDonald, K. Balachandran, R. Singh, S. Garg, H. Desai, K. Davies, W. Goddard; Blackpool: G. Galasko, I. Rahman, Y. Chua, O. Payne, S. Preston, O. Brennan, L. Pedley, C. Whiteside, C. Dickinson, J. Brown, K. Jones, L. Benham, R. Brady; Carlisle: L. Buchanan, A. Ashton, H. Crowther, H. Fairlamb, S. Thornthwaite, C. Relph, A. McSkeane, U. Poultney, N. Kelsall, P. Rice, T. Wilson; Chertsey: M. Wrigley, R. Kaba, T. Patel, E. Young, J. Law; Cramlington: C. Runnett, H. Thomas, H. McKie, J. Fuller, S. Pick; Exeter: A. Sharp, A. Hunt, K. Thorpe, C. Hardman, E. Cusack, L. Adams, M. Hough, S. Keenan, A. Bowring, J. Watts; Great Yarmouth: J. Zaman, K. Goffin, H. Nutt; Harrogate: Y. Beerachee, J. Featherstone, C. Mills, J. Pearson, L. Stephen- son; Huddersfield: S. Grant, A. Wilson, C. Hawksworth, I. Alam, M. Robinson, S. Ryan; Macclesfield: R. Egdell, E. Gibson, M. Holland, D. Leonard; Maidstone: B. Mishra, S. Ahmad, H. Randall, J. Hill, L. Reid, M. George, S. McKinley, L. Brockway, W. Milligan; Manchester: J. Sobolewska, J. Muir, L. Tuckis, L. Winstanley, P. Jacob, S. Kaye, L. Morby; Nottingham: A. Jan, T. Sewell; Poole: C. Boos, B. Wadams, C. Cope, P. Jefferey; Portsmouth: N. Andrews, A. Getty, A. Suttling, C. Turner, K. Hudson, R. Austin, S. Howe; Redhill: R. Iqbal, N. Gandhi, K. Brophy, P. Mirza, E. Willard, S. Collins, N. Ndlovu; Rhyl: E. Subkovas, V. Karthikeyan, L. Waggett, A. Wood, A. Bolger, J. Stockport, L. Evans, E. Harman, J. Starling, L. Williams, V. Saul; Salisbury: M. Sinha, L. Bell, S. Tudgay, S. Kemp, J. Brown, L. Frost; Shrewsbury: T. Ingram, A. Loughlin, C. Adams, M. Adams, F. Hurford, C. Owen, C. Miller, D. Donaldson, H. Tivenan, H. Button; South Shields: A. Nasser, O. Jhagra, B. Stidolph, C. Brown, C. Livingstone, M. Duffy, P. Madgwick; Southampton: P. Roberts, E. Greenwood, L. Fletcher, M. Beveridge, S. Earles; Taunton: D. McKenzie, D. Beacock, M. Dayer, M. Seddon, D. Greenwell, F. Luxton, F. Venn, H. Mills, J. Rewbury, K. James, K. Roberts, L. Tonks; Torquay: D. Felmeden, W. Taggu, A. Summerhayes, D. Hughes, J. Sutton, L. Felmeden; Watford: M. Khan, E. Walker, L. Norris, L. O’Donohoe; Weston-super-Mare: A. Mozid, H. Dymond, H. Lloyd-Jones, G. Saunders, D. Simmons, D. Coles, D. Cotterill, S. Beech, S. Kidd; Wolverhampton: B. Wrigley, S. Petkar, A. Smallwood, R. Jones, E. Radford, S. Milgate, S. Metherell, V. Cottam; Yeovil: C. Buckley, A. Broadley, D. Wood, J. Allison, K. Rennie, L. Balian, L. Howard, L. Pippard, S. Board, T. Pitt-Kerby.*

*APHRS-AF Registry Steering Committee (National Coordinators)*:

*Hong Kong: Chun-Wah Siu David (Queen Mary Hospital). Japan: Wataru Shimizu, Kenji Yodogawa (Department of Cardiovascular Medicine, Medical School); Hiroyuki Tsutsui, Yasushi Mukai (Department of Cardiovascular*

*Medicine, Faculty of Medical Sciences, Kyushu University); Hirofumi Tomita, Daisuke Horiuchi (Department of Cardiology, Hirosaki University Graduate School of Medicine); Joji Hagii (Hirosaki Stroke and Rehabilitation Center); Kazutaka Aonuma (Division of Cardiology, University of Tsukuba Hospital); Yasuo Okumura (Division of Cardiology, Nihon University, Itabashi Hospital); Masahiko Goya, Kenzo Hirao (Department of Cardiovascular Medicine, Tokyo Medical and Dental University); Ajioka (Division of Cardiology, Tosei General Hospital); Nobuhisa Hagiwara, Atsushi Suzuki (Department of Cardiology, Tokyo Women’s Medical University); Teiichi Yamane (Department of Cardiovascular Medicine, Jikei University); Takanori Ikeda, Hitomi Yuzawa (Toho University, Faculty of Medicine); Kazuhiro Satomi, Yoshinao Yazaki (Heart Rhythm Center, Tokyo Medical University); Keiichi Fukuda (Department of Cardiology, Keio University School of Medicine); Yoshinori Kobayashi, Norishige Morita (Division of Cardiology, Tokai University Hachioji- hospital); Toyoaki Murohara (Department of Cardiology, Nagoya University); Eiichi Watanabe, Masahide Harada (Department of Cardiology, Fujita Health University School of Medicine); Satoru Sakagami, Takahiro Saeki (National Hospital Organization, Kanazawa Medical Center); Kengo Kusano, Koji Miyamoto (Department of Cardiovascular Medicine, National Cerebral and Cardiovascular Center); Shinsuke Miyazaki, Hiroshi Tada (Department of Cardiovascular Medicine, University of Fukui); Koichi Inoue, Nobuaki Tanaka (Cardiovascular center, Sakurabashi Watanabe Hospital); Yukihiro Koretsune, Haruhiko Abe (National Hospital Organization Osaka National Hospital); Yasuki Kihara, Yukiko Nakano (Department of Cardiovascular Medicine, Hiroshima University); Akihiko Shimizu, Yasuhiro Yoshiga (Department of Medicine and Clinical Science, University Graduate School of Medicine); Tomohiro Sakamoto, Ken Okumura (Division of Cardiology, Saiseikai Kumamoto Hospital Cardiovascular Center); Naohiko Takahashi, Tetsuji Shinohara (Oita University Hospital); Kyoko Soejima (Department of Cardiovascular Medicine, Kyorin University School of Medicine); Masahiko Takagi (Kansai Medical University Medical Center); Mitsuharu Kawamura, Yumi Munetsugu (Division of Cardiology, Showa University School of Medicine). Korea: Sung- Hwan Kim (Division of Cardiology, The Catholic University of Korea); Jae-Min Shim (Division of Cardiology, Korea University College of Medicine and Korea University Medical Center); Jae Sun Uhm (Division of Cardiology, Yonsei University College of Medicine); Sung Il Im (Division of Cardiology, Kosin University College of Medicine); Hyoung-Seob Par (Division of Cardiology, Department of Internal Medicine, Keimyung University Dongsan Hospital); Jun Hyung Kim (Department of Cardiology, Chungnam National University); Young Keun On (Division of Cardiology, Sungkyunkwan University School of Medicine); Il-Young Oh (Division of Cardiology Seoul National University Bundang Hospital); Seung Yong Shin (Cardiovascular & Arrhythmia Centre, Chung-Ang University); Jum Suk Ko (Division of Cardiology, Department of Internal Medicine, Wonkwang University School of Medicine, Iksan, Korea); Jun Beom Park (Department of Cardiology, College of Medicine, Ewha Womans University, Seoul, Korea). Singapore: Wee-Siong Teo (National Heart Centre Singapore); Kelvin Cheok-Keng Wong (Changi General Hospital); Toon-Wei Lim (National University Hospital); David Foo (Tan Tock Seng Hospital). Taiwan: Shih-Ann Chen (Taichung Veterans General Hospital); Shih-Ann Chen, Tze-Fan Chao, YennJiang Lin, Fa-Po Chung, Yu-Feng Hu, Shil-Lin Chang, Ta-Chuan Tuan, Jo-Nan Liao (Taipei Veterans General Hospital); Cheng-Hung Li, Jin-Long Huang, Yu-Cheng Hsieh, Tsu- Juey Wu, Ying-Chieh Liao (Taichung Veterans General Hospital); Cheng- Hung Chiang, Hsiang-Chiang Hsiao, Tung-Chen Yeh (Kaohsiung Veterans General Hospital); Wei-Siang Lin, Wen-Yu Lin (Tri-Service General Hospital); Jen-Yuan Kuo, Chong-Lie Hong, Yih-Je Wu, Ying-Siang Li, Jui-Peng Tsai,*

*Kuo-Tzu Sung, Sheng-Hsiung Chang (Mackay Memorial Hospital).*
